# ‘Now I care’: a qualitative study of how overweight adolescents managed their weight in the transition to adulthood

**DOI:** 10.1136/bmjopen-2015-010774

**Published:** 2016-11-02

**Authors:** Helen Sweeting, Emily Smith, Joanne Neary, Charlotte Wright

**Affiliations:** 1 MRC/CSO Social and Public Health Sciences Unit, Institute of Health and Wellbeing, University of Glasgow, Glasgow, UK; 2 University Hospital Southampton NHS Foundation Trust, Southampton, UK; 3 Department of Public Health, Institute of Health and Wellbeing, University of Glasgow, Glasgow, UK; 4 School of Medicine, University of Glasgow, Glasgow, UK

**Keywords:** adolescence, overweight, weight management, weight loss, QUALITATIVE RESEARCH

## Abstract

**Objectives:**

A qualitative study of recalled experiences of early adolescent overweight/obesity revealed low levels of weight-related concern. This further analysis aimed to explore weight-related concern and weight-loss efforts as participants transitioned into adulthood.

**Design, participants and methods:**

Participants were 35 young adults from a population-based cohort study who had body mass index (BMI) >95th centile between ages 11 and 15 and participated in semistructured interviews aged 24. At age 24, they were categorised as: ‘*slimmers*’ (N=13) who had lower BMI Z-scores at 24 than their adolescent peak and were not obese (BMI<30 kg/m^2^); ‘*relapsers*’ (N=8, of whom 2 were morbidly obese (BMI>35 kg/m^2^) at age 24); ‘*stable*’ (N=3, of whom 1 morbidly obese); and ‘*gainers*’ (N=11, of whom 5 morbidly obese). Themes were identified and coded using NVivo qualitative data analysis software, blind to participants’ current weight status.

**Results:**

Contrasting with the lack of concern recalled in respect of earlier adolescence, weight-related concerns and/or desire to lose weight generally increased around the time of school leaving and almost all participants described some form of exercise (formal/informal) and dietary weight-control strategies. Among ‘slimmers’, there was some (subtle) evidence of more consistent use of exercise, self-monitoring of diet and exercise and of lifestyle changes becoming habitual and/or part of identity. Few participants had accessed professional support. Diet clubs seemed to have been used most by ‘gainers’, some only recently. Labour-market and housing transitions were strong influences, described as facilitating weight losses by some, but increases by others. For some participants, it appeared that weight loss was simply a by-product of these transitions.

**Conclusions:**

In contrast to earlier adolescence, even the heaviest participants tended to show actual weight loss action or preparation for action. The transition to adulthood could thus be a key life stage for interventions.

**Strengths and limitations of this study**

- This is one of very few qualitative studies, and the first in the UK, to explore reasons why overweight community-based adolescents do or do not lose weight, in the transition to adulthood.
- It subsampled from a longitudinal study with measured body mass index (BMI) at several points in adolescence, enabling objective categorisation of BMI changes over time.
- Our findings resulted from secondary analysis of qualitative data from a study which did not specifically set out to identify some of the highlighted themes.
- Not all adolescent weight changes described by participants were detected by our measurement schedule (eg, some described losing then regaining weight in the years between) and some categorised as ‘slimmers’ had experienced BMI increases since their very lowest point.

## Introduction

Adolescent overweight is associated with greatly increased likelihood of adult obesity,^1^ but up to a third of obese adolescents do not go on to be obese adults.^2^ What is not clear is why and how some overweight/obese adolescents (defined broadly, by the WHO, as those aged 10–19^3^) lose weight and others do not,^4^ and why some adolescents maintain weight loss while others regain weight. The few population-based studies that have examined this question have generally found very limited or inconsistent behavioural differences between adolescent weight losers, gainers and/or maintainers. A national US survey of adolescents found differences in physical activity, but none in reported diet.^5^ Two smaller US and New Zealand studies found ‘healthful’ dietary and PA behaviours, and self-monitoring were associated with loss, but specific dietary plans were not^6^ ^7^ and a study of female Swedish adolescents found no clear behavioural differences.^8^ Analysis of the Scottish cohort from which participants in the present study were drawn found no differences in reported dieting at either 11 or 15 between the continually obese and those who had slimmed.^9^

Active initiation of weight loss behaviours requires that overweight/obesity is recognised and perceived as a problem.^10–13^ Community-based quantitative studies have shown obese children and adolescents tend to have more negative body image than non-obese peers.^14^ ^15^ However, a qualitative UK study found high acceptance of body size among disadvantaged overweight/obese Scottish 13–14 years,^16^ while qualitative US and Australian studies have found that adolescents recognise obesity as a societal issue, but not in themselves, and thus have low motivation to implement behavioural changes.^17–19^

One reason why so little is known about how young adults view overweight/obesity or approach weight loss is that while it is relatively easy to study adolescents ‘captive’ in school or treatment programmes, they become largely invisible to researchers once they leave school. We have previously reported findings from a qualitative study of young adults nested within a large Scottish cohort study, where participants were well documented as having been overweight/obese in adolescence. This revealed widespread recalled recognition of, but lack of major recalled concern about their overweight/obesity during adolescence,^20^ consistent with other studies.^16–19^ This data set also included reflections by participants on their lives since adolescence which have not so far been reported. Thus, this new qualitative analysis aimed to explore their transition to adulthood, by examining postschool weight-related concerns, behaviours and experiences related to young adult transitions among 24 year-olds categorised in terms of measured adolescent body mass index (BMI) trajectories and current BMI. Specifically, we considered whether they described more concern about their overweight/obesity in the postschool transition to adulthood and how other aspects of their late adolescent/young adult lives impacted on their weight-related behaviours.

## Methods

### Participants and recruitment

In 2008, 35 young adults (age 24; 17 males; 33 White Scottish and 2 mixed/Asian ethnicity) participated in a study of recalled adolescent experiences of obesity conducted by ES.^21^ They were purposively subsampled from the longitudinal *West of Scotland 11–16/16+*Study, which obtained data from school pupils at age 11 (N=2586; 93% of issued sample), 13, 15^9^ and 19 (N=1256). Height and weight measurements were taken at each stage, allowing calculation of BMI and BMI z-scores^22^ and identification of participants with BMI z-scores >95th centile compared with British 1990 growth references^22^ between ages 11 and 15, as described previously.^20^ ^21^

University of Glasgow ethical approval was obtained for the qualitative substudy and participants completed consent forms, including permission to publish anonymised extracts from their interviews.

### Interviews

Semistructured interviews were conducted by ES and audio-recorded with consent. They began with a picture task designed for this study (paired images of young people with a range of BMIs and diet/exercise behaviours) to stimulate discussion around perceptions of bodies and health. Next, participants were asked to describe themselves and their lives as a teenager and changes as they became young adults. These generally prompted discussion of postschool labour market transitions, health, concerns (including in relation to health/weight), interests and activities, eating patterns and relationships/support (see online supplementary file 1—interview topic guide and example picture task items).

10.1136/bmjopen-2015-010774.supp1 supplementary file

### Analyses

The interviews were transcribed verbatim and pseudonyms applied. For this paper, a secondary analysis approach was taken. Previous analyses^20^ ^21^ meant ES, HS and CW were already familiar with the data set. A researcher with no prior knowledge of the study (JN) familiarised herself with the transcripts, then, with HS and CW, identified themes relating to postschool late adolescent/young adult experiences. Themes, identified deductively (based on previous literature) and inductively (from the data), were coded by JN using NVivo qualitative data analysis software, blind to participant BMI. These were then reviewed with HS and CW and checked against transcripts by HS during write-up of the paper. Separately, CW converted all adolescent BMIs into age-specific and gender-specific Z-scores compared with the UK 1990 reference.^23^ ^24^ Age 24 BMIs were converted to Z-scores for age 19.99, the highest age of the reference. She then categorised participants into one of four relatively homogenous BMI trajectory groups on the basis of adolescent (age 11–15) and young adult BMI: ‘*slimmers*’ had lower BMI Z-scores at age 24 than their adolescent peak and were not obese (BMI<30 kg/m^2^); ‘*relapsers*’ had shown a decrease from their peak adolescent BMI Z-score, followed by regain and were currently obese; the BMI Z-scores of ‘*stable*’ participants had remained largely unchanged throughout; finally, the BMI Z-scores of ‘*gainers*’ had steadily increased since adolescence.

This paper describes the most common themes raised by participants in relation to late adolescence/young adulthood (weight-related concerns; exercise; diet; professional support; young adult transitions) and relates these to their adolescent/young adult BMI categories.

## Results

Table 1 shows 13 participants were ‘slimmers’, of whom 3 were of normal weight (BMI<25 kg/m^2^) and 10 were overweight at age 24. Eight were ‘relapsers’ (6 obese, 2 morbidly obese—BMI>35 kg/m^2^), 3 were ‘stable’ (1 overweight, 1 obese, 1 morbidly obese) and 11 were ‘gainers’ (6 obese, 5 morbidly obese). Individual graphs show considerable variations in BMI trajectory (see online supplementary file 2—individual BMI Z-score trajectories). Some participants also described weight changes not apparent in these study measurements and discussed the circumstances in which they had occurred.

**Table 1:** Participants categorised according to current body mass index (BMI), change since highest BMI in adolescence and whether had slimmed and relapsed previously

	As adolescent (ages 11–15)		As young adult (age 24)		
Pseudonym	Peak adolescent BMI Z-score	Age of max adolescent BMI	BMI	BMI Z-score	BMI category
‘*Slimmers’ (M=*6*, F=*7*)*					
Lower BMI than in adolescence and not obese as young adult					
Catherine	2.37	15	24.0	0.55	Normal
Nina	1.82	13	23.8	0.49	Normal
Noel*	2.46	15	20.7	−1.04	Normal
Alan	2.06	13	27.4	1.33	Overweight
Charlie	2.90	15	28.7	1.64	Overweight
Clare	1.94	11	27.1	1.39	Overweight
Eilidh	3.08	13	29.3	1.88	Overweight
Emma	2.37	11	28.4	1.68	Overweight
Janine	2.37	15	25.4	0.95	Overweight
Mark	2.18	15	29.5	1.83	Overweight
Pete	2.17	15	28.0	1.47	Overweight
Rachel	1.71	15	26.1	1.14	Overweight
Scott	2.38	11	26.5	1.07	Overweight
*‘Relapsers' (M=*5*, F=*3*)*					
Obese, slimmed previously					
Colin	2.68	15	30.2	1.98	Obese
Laura	1.66	15	30.2	2.06	Obese
Malcolm	2.02	15	30.8	2.09	Obese
Patricia	2.82	13	33.1	2.55	Obese
Patrick	1.66	15	30.5	2.04	Obese
Philip	1.88	11	30.4	2.01	Obese
Donna	3.24	15	37.8	3.20	Morbidly obese
Geoff	3.14	13	44.3	3.87	Morbidly obese
‘*Stable’ (M=*1*, F=*2*)*					
No change since adolescence					
Chris	1.90	13	29.6	1.84	Overweight
Christina	2.55	15	32.4	2.44	Obese
Jenny	3.24	15	38.3	3.25	Morbidly obese
*‘Gainers’ (M=*5*, F=*6*)*					
Increased obesity since adolescence					
Jamie	2.37	15	†	2.57†	Obese
Matthew	1.91	15	32.9	2.49	Obese
Michael	1.88	13	31.9	2.31	Obese
Natasha	2.03	15	32.0	2.37	Obese
Neil	2.06	13	34.9	2.75	Obese
Sarah	2.28	15	32.7	2.49	Obese
Anne	3.64	15	43.1	3.74	Morbidly obese
Elizabeth	3.21	15	41.2	3.56	Morbidly obese
Kirsty	2.65	15	43.5	3.78	Morbidly obese
Lisa	3.19	15	‡	3.79‡	Morbidly obese
Richard	2.84	15	42.7	3.71	Morbidly obese

*Weight loss attributed by participant to severe postviral illness at age 17.

†Did not consent to be weighed at age 24 but observed to be obese; age 19 BMI Z-score provided.

‡Did not consent to be weighed at age 24 but observed to be extremely obese; age 19 BMI Z-score provided.

10.1136/bmjopen-2015-010774.supp2 supplementary file

### Weight-related concerns

Most participants, regardless of BMI trajectory group, described increasing weight-related concerns and/or desire to lose weight as they progressed into later adolescence (table 2; see online supplementary table S1 for extensive illustrative quotes). Several related their increasing concerns to a wish for a new identity as part of the postschool transition; Eilidh ‘*realised that I was going to uni, I didn't want to be big, it was like a new kinda fresh start*’. However, most described their attitudinal change in terms of more general maturity and acknowledgement of weight as personal responsibility. Examples among ‘slimmers’ included Janine, who became ‘*conscious*’ of her weight around age 15–16 and Mark who noted ‘*it was only in my late teens that I started to be aware of this concept of healthy living, yeah, it wasn't something that ever kinda touched me as a, as a fifteen year-old boy*’. Malcolm (‘relapser’) ‘*left school thinking “nah, I don't care about dieting” … and then that kinda stopped and I was like that, “oh wait a minute, need to try and do something*”’. Such accounts were also evident among ‘gainers’: Anne said that ‘*as I got older I realised that I had to do something*’, Elizabeth had ‘*changed since I've been a teenager, because I watch what I'm eating*’ and Sarah, who was ‘*finally on a diet for the first time properly in my life*’ described herself as ‘*far more mature than I used to be*’. There was no evidence that increasing concern was limited to those who had at some stage lost weight, apart from hints that perhaps non-‘slimmers’ expressed concerns in slightly vaguer terms and, for a small number, they appeared to have occurred more recently.

**Table 2:** Illustrative quotes according to participant ‘slimmer’, ‘relapser’, ‘stable’ and ‘gainer’ categorisation—weight-related concerns

Slimmer	
Eilidh	Yeah I don't know I think when I started coming to the end of high school and realised that I was going to Uni, I didn't want to be big, it was like a new kinda fresh start Once I got to a size 16 I just got kinda lazy and went ‘well, I'm fine now’, do you know. I'd, I would like to lose a wee bit more but I'm quite content the way I am do you know
Relapser	
Malcolm	I still left school thinking, ‘nah I don't care about dieting’, again ‘if I eat I'm just gonna burn it off, quicker than anyone else’ and then that kinda stopped and I was like that ‘oh wait a minute, need to try and do something’
Stable	
Christina	But I'm quite vain, even though I'm big, I think I'm shit hot, do you know what I mean? … I am quite vain, even though there's things that I would like tae change, but I'm no gonna bust a gut tae change them, do you know what I mean?
Gainer	
Sarah	I am now finally on a diet for the first time properly in my life, so I've joined Weight Watchers a couple of months ago so I've now lost just over a stone … so I'm finally trying to do something about it cos it bothers me

10.1136/bmjopen-2015-010774.supp3 supplementary tables

However, some participants expressed current acceptance of their size. For example, Geoff (‘relapser’) was not ‘*overly concerned*’, having decided ‘*this is what I'm are*’ [sic], Christina (‘stable’) described herself as ‘*quite vain, even though I'm big, I think I'm shit hot’* and Jenny (‘stable’) did not want to ‘*go to all these classes to get healthy. As long as I don't feel like crap I'm not too bothered like*’. Two ‘slimmers’ expressed acceptance only once they felt more comfortable with their clothes size. Eilidh described herself as becoming ‘*lazy’* and ‘*content*’ on reaching size 16, and Rachel ‘*realised as I got older that I was never supposed to be a size six or a size eight, that's just not the way I'm built*’.

### Exercise

In response to these concerns, almost all participants described behavioural changes, including diet (next section) and exercise, particularly in gyms, but also team sports, swimming, use of home exercise DVDs/gym equipment, running and walking (table 3; see online supplementary table S2).

**Table 3:** Illustrative quotes according to participant ‘slimmer’, ‘relapser’, ‘stable’ and ‘gainer’ categorisation—exercise and diet

Slimmers	
Mark	I don't remember the moment of making the decision, but I do remember coming home from school and getting changed and going to the gym and that was, that was very… it was a bit of a departure from the way life was for me before then … it became part of my life and it has remained so to this day
Catherine	So aye, it was losing the weight, it was, it was hard at the start, but see once you get into a routine of knowing what you do, what you can eat, what you can't eat, what you need to keep yourself away fae, it is quite easy
Relapsers	
Geoff	When I left school I went to I done, I done boxing, fitba, I went to the gym. … I wis I say I wis playing aw the sports. So if I could eat that but I, I wisny putting on any weight cos I wis going to the gym, playing fitba and that. I don't play a lot o’ fitba noo right enough. I'd like tae but it's getting the time and the people tae play it
Laura	Maybe in the last couple of years or so, in the sense that, yeah, you go out and do lunches with your friends and this and that, and you think that I could really do with cutting some of that out. You know, weekend fry-ups and stuff like that. Trying to be healthier and, you know, the healthy option …
Stable	
Chris	I never did anything particularly excessive. I never did anything too… you know, tried… sort of stuck to anything very long I don't think when I was, when I was younger, so I guess that's probably why nothing ever worked
Jenny	I can just eat really good foods and be really good but it never makes that much of a difference
Gainers	
Anne	I used to go to the gym on a Monday but it's shut now, the gym that I go to, it's not opened anymore. Em, for refurbishment. But like, I've got like exercise DVDs now that I'll do in the house
Jamie	Just cut out junk, I cut out a lot of carbs I remember… Yeah it was that what I did I remember doing, I remember saying ‘no junk’… You really do need a disciplined and healthy eating plan. You know says the man who had a bag of crisps and a Mars Bar last night …

Most ‘slimmers’ mentioned the gym. Pete and Mark started attending while still at school, which for Mark was ‘*a bit of a departure from the way life was for me before*’. Scott's, Charlie's, Claire's and Rachel's gym attendance began at university. Charlie found it ‘*wasn't even difficult*’ and this ‘*total change in lifestyle*’ resulted in weight loss. Claire used the gym ‘*throughout my uni life*’, and Rachel managed gym attendance, university classes and bar work. Exercise had been sustained by all this group. For Mark, the gym environment ‘*became part of my life and has remained so to this day*’, Scott continued to ‘*train hard*’ and Charlie described how ‘*now I jist sorta sustain*’ exercise. Claire's exercise had become ‘*kind of habit … I don't think I have to go to the gym or do this, to exercise I would just do, walking, jogging, whatever*’ and Rachel went ‘*to the gym a lot*’. Among the other ‘slimmers’, Emma's police training involved time at the gym, circuits and swimming and was ‘*the most active I think I have ever been in my life*’; she also continued to attend. Eilidh and Catherine had tried a gym, but preferred other activities; Eilidh ‘*loved’* cycling and Catherine walked with her baby buggy. While acknowledging impact on weight, Nina and Noel were vaguer about their exercise.

Some ‘relapsers’ linked weight loss to exercise. At around 17–18, Patricia ‘*lost a drastic amount of weight … and I was exercising an awful lot*’, Colin had a ‘*fitness freak stage*’ and Geoff found he could maintain his weight by balancing eating with exercise. However, only Patricia's gym attendance continued. Exercise featured less in the accounts of other ‘relapsers’, including Malcolm, for whom ‘*there's not been any exercise really, not much*’, Laura, who occasionally used a home trampoline, although ‘*there's just those weeks when you can't be bothered*’, and Donna who had recently tried to increase her exercise via walking. Similarly, Chris (‘stable’) thought not sticking with anything was ‘*probably why nothing ever worked*’ while Christina who regularly walked her dog ‘*wouldnae go tae a gym*’.

In exactly the same way, several male ‘gainers’ described earlier periods of significant exercise which had ceased for reasons, including the need to focus on academic work, injuries, lack of time or motivation. Some female ‘gainers’ described exercising: Anne had attended a gym which was now closed, but used home exercise DVDs, Elizabeth had discovered aqua-aerobics and Kirsty had recently joined a gym.

### Diet

Participants tended to discuss diet in two ways. First, the importance of having a balanced diet that used home cooking rather than relying on frozen/take-away meals, with healthy choices such as less cheese or cream-based sauces and more fruit. Second, they described their experiences of participating in calorie-controlled diets, either as promoted by commercial slimming clubs or unsustainable ‘fad’ diets (eg, liquid diets, drinking vinegar, avoiding dairy/gluten/carbohydrates or foods of a particular colour) (table 3; see online supplementary table S3).

Several female ‘slimmers’ related their weight loss to reduced food intake and meal-skipping: Rachel ‘*just changed the way I ate*’. Many ‘slimmers’ described the need to be constantly mindful of food choices: Mark had not bought certain foods in order to control his intake; Scott self-monitored, ‘*there's times whereby I'll pick up a biscuit and I'll go “no, I don't want it”’*; Nina noted ‘*the [weight-related] worrying's definitely stayed there*’; and Eilidh described herself as ‘*very, very always watching about not getting bigger*’. However, some appeared slightly more relaxed, including Catherine who described ‘*a routine of knowing what you do, what you can eat, what you can't eat, what you need to keep yourself away fae. It is quite easy*’.

A similar range of strategies was described by participants in the other groups, but with perhaps less emphasis on real and sustainable reductions in intake or continued vigilance. Among the ‘relapsers’, Patricia had lost weight by meal skipping, Donna had achieved weight loss via severe dieting but now ate ‘healthy’ food, while one of Colin's adolescent weight-loss strategies had been to make himself sick; this had stopped and he was trying to ‘*eat something a bit more healthier*’. Malcolm believed controlling food intake was more important than exercise for weight loss, but did so by skipping breakfast. He and Philip talked about home-cooked meals while Laura mentioned ‘*you know, the healthy option*’. Christina (‘stable’) noted that ‘*I dae eat quite healthily but it's my amounts*’; she had unsuccessfully tried a range of ‘fad’ diets. However, Jenny (stable) believed ‘*I can just eat really good foods and be really good but it never makes that much of a difference*’.

Two ‘gainers’, Sarah and Kirsty, had recently started seriously dieting, using commercial slimming club regimes. Elizabeth reported losing weight when on a commercial club diet, and was currently focusing on ‘*watch[ing] what I'm eating*’, but Anne believed dieting had caused stomach problems so ‘*I'd had to eat things to suit my stomach, rather than suit my diet*’. Lisa also reported losing weight via a commercial club, but it increased once she ‘*stopped recording things and checkin*g’. Although more often described by females, a small number of male ‘gainers’ also described dieting: Michael had reduced his calorie intake on the advice of his GP, and Richard ‘*didn't have a takeaway for six mon*ths’, but then, to use Jamie's description, his diet went ‘*a bit awry again*’.

### Professional support

Contrasting with self-initiated and/or unsupported behavioural changes, professional support (eg, slimming clubs, fitness classes, GP advice) was mentioned by very few participants (table 4; see online supplementary table S4). Only one ‘slimmer’, Pete, mentioned that at around age 19–20, he had asked his GP and been helped by simple advice on portion control, exercise and social support. Patricia (‘relapser’) reported her GP had told her ‘*och it's OK you don't need to lose weight*’. She had also attended a council-run weight-management service, Weight-Watchers and used a personal trainer.

**Table 4:** Illustrative quotes according to participant ‘slimmer’, ‘relapser’, ‘stable’ and ‘gainer’ categorisation—professional support

Slimmer	
Pete	I went to you know like my GP a couple of times to try and get advice on how to, you know what I should do. … [was advised] just to try and control portions and try to, to count, you know not count calories but be mindful of what the intake was and perhaps to, to exercise regularly you know with, either with friends or you know try and get support you know. So that did help a lot. That did help
Relapser	
Patricia	I was referred to the Council's weight-management service by my doctor, and I went and never lost any weight there, and because I never lost any weight, they just never got back in contact. And my doctor I feel because she's so big, when I go and I say ‘I would really, really like to lose weight and I'll, I can show you a food diary of what I've been eating, I can show you my exercise, I can show you how much water I've been drinking’, my doctor will go, ‘och it's ok you don't need to lose weight’
Gainers	
Lisa	I went to Weight Watchers classes and lost a good bit of weight … the reason I left was a lot of it was getting me down because, em, there was too much emphasis on figures, like you've lost or you've gained or you're this or you're that
Richard	My cousin dragged me tae Weight Watchers. … It's actually alright. I liked it. I went for aboot four months … I've got a family doctor … She's always geeing me an earful to get oan at me, and every time I go up that's the first thing she does. If I go up for a sore throat she weighs me, so she's always on my back to get me to lose weight. … So I've no been up for aboot eight month noo, coz I'm terrified of going up again in case she shouts at me again

Similar, if not more, professional input was mentioned by ‘gainers’, some describing this as helpful. Anne spoke vaguely about ‘slimming clubs’, but Lisa lost ‘*a good bit of weight*’ via 2 years' Weight-Watchers attendance. Richard reported losing around 15 kg, having been ‘*dragged*’ to Weight-Watchers. However, he subsequently regained the weight and stopped attending his GP because ‘*She's always geeing me an earful to get oan at me, and every time I go up that's the first thing she does. If I go up for a sore throat she weighs me, so she's always on my back to get me to lose weight*’. Similarly, Michael reported his GP said ‘*if I keep cerry on the way I was, I was gonna have a heart attack by the time I was thirty-five, and that put the shitters right up me*’. However, he found her simple dietary and exercise advice useful. Two ‘gainers’ had started attending slimming clubs only very recently, with Kirsty reporting that ‘*I'm ready to take that step to lose weight*’.

### Young adult transitions

Participants had experienced a range of young adult transitions: 23 had attended tertiary education in the past (university and college, including college-based apprenticeships) and 4 were doing so at the time of the interview; 29 were working and 5 had performed so in the past; 19 were living in their own homes and 3 had left the parental home in the past but were living back there at the time of the interview; 1 was a parent. These young adult transitions (which were broadly similar across BMI trajectory groups) appeared key to weight changes for many participants, regardless of BMI trajectory group (table 5; see online supplementary table S5). Thus, across the groups, some described college/university as a fresh start and/or facilitator to exercise which then meant they met active peers. A few learnt about nutrition or PA, enabling reflection on personal choices. However, others felt college/university was connected with weight-gain, mainly via poor diet and alcohol. Employment was also described as both facilitating and impeding weight loss. Several described loss resulting from active jobs and a few used their earnings to join a gym. However, others worked in sedentary jobs, felt too exhausted by work to bother with home cooking or exercise, or spent their earnings on ‘junk’ food and alcohol. Leaving home was also linked to increased dietary control and so healthier options for some but less balanced meals for others; the small number living with a partner described this as increasing the likelihood of home-cooking.

**Table 5:** Illustrative quotes according to participant ‘slimmer’, ‘relapser’, ‘stable’ and ‘gainer’ categorisation—young adult transitions

Slimmers	
Catherine	WORK: I changed my jobs in August last year, and since then, the amount of weight I have lost is unbelievable. I think I've lost about a stone and a half since August … it's just through daen more, being more active, than compared to what I was doing
Scott	EDUCATION: The lifestyle wasn't so much a big thing about until I turned maybe eighteen, nineteen and started doing my degree then I started learning how to use a gym properly and what sort of exercise that I can do and just I'm now very aware of cos I'm working in nutrition what it is I actually take in and what it is I actually expend
Relapsers	
Donna	EDUCATION/LEAVING HOME: That wasn't actually so much of a help because I was living on my own. At student houses and everything else and takeaways was a much more tempting option than cooking for yourself more often than not. Again throughout my Uni career, first to fourth year, I gradually, I definitely improved. I got a grip of that and decided that eating healthy was, was the best option so I started cooking for myself
Stable	
Chris	EDUCATION/LEAVING HOME: When I was at uni and I joined the gym and pretty much spent all the money I had on cigarettes and alcohol and didn't eat as much as probably I should have, but not in a you, know, not in a deliberate way, just like I used to never have any money for food and so I lost quite a lot of weight then
Gainers	
Jamie	EDUCATION: There was first, first and second year at Uni when I just, you know I discovered you know booze. And then that really was us off to the races in terms of overweight
Neil	WORK: I was labouring for a wee while. I must have laboured for about six months. … I didn't try to lose weight, when I started the job, I didn't try to lose weight, initially, at all—it didn't enter my mind. … then it became, for me, at my work, at my workplace, where I could be getting paid for losing weight, basically

Among the ‘slimmers’, Charlie, Clare, Mark and Scott all described weight loss associated with attending university. Charlie's close friends also went to the gym, while Mark was encouraged by a coach; for him ‘*coming to uni was the sort of the biggest change ever*’. When Eilidh started university, she ‘*just started really healthy eating*’ and took up swimming. Catherine and Scott's courses involved nutrition, with Catherine noting ‘*it kinda opens your eyes to things that you're eating and what it is doing to you*’. Weight loss was a requirement for Alan's admission to the RAF and Emma's police job, and their subsequent training involved PA. Both had maintained weights well below the adult obesity level, but Emma described consciously relaxing her regime since achieving her goal of becoming a police officer. Janine had worked as a show dancer, which required physical fitness, but also encouraged high levels of social drinking, ‘*so it was a bit of both—bad and good*’. Catherine had recently left a job at a fast food counter and ‘*the amount of weight I have lost is unbelievable*’.

‘Relapsers’ and those for whom our measurements showed ‘stable’ BMIs provided largely similar accounts. Patricia and Chris described losing weight at university, Patricia by meal-skipping attributed to a busy routine and Chris because he ‘*pretty much spent all the money I had on cigarettes and alcohol and didn't eat as much as probably I should have*’. Donna dealt with university workload stress by eating, and in student accommodation ‘*takeaways was a much more tempting option than cooking for yourself*’. Although several ‘relapsers’ mentioned gym attendance, Chris was the only one who linked this with university. Philip lost weight after leaving school without conscious effort because ‘*I was working full-time. … I wasn't able to go to like Gregg's [bakers] twice a day and stuff like that*’. Christina thought she had lost weight ‘*by accident*’ due to stress and other changes involved in moving into her own home, while living with a friend/partner had forced Malcolm and Philip to begin home cooking.

Weight loss facilitated by young adult transitions was also mentioned by some ‘gainers’: Jamie attended the gym and dieted during his third university year and that was ‘*probably the best shape I was in*’ and Richard attributed weight loss at college to football and gym attendance. Neil found he ‘*could be getting paid for losing weight*’ while working as a building labourer for 6 months. He also ascribed weight fluctuations to his relationship status: ‘*whenever I meet a lassie I'll be in tip top condition and then, within a year I've put on like a stone and a half’.* Sarah thought her current nursing job meant ‘*I can't really preach healthy living to people if I'm not actually doing it myself*’. However, accounts in this group also tended to describe transition-related barriers to weight loss. Jamie ‘*discovered booze*’ at university ‘*and then that really was us off to the races in terms of overweight*’. Other ‘gainers’ described the impact of shift-work, on diet (‘*no eating breakfast again, and grabbing a bar of chocolate*’—Kirsty) and motivation to exercise (‘*after a day's work I'm absolutely knackered and I don't want to go out for a run*’—Matthew).

## Discussion

Studies that track from adolescence into adulthood are relatively rare. In our sample of previously overweight or obese adolescents, over a third had not gone on to become obese adults, but almost a quarter were already morbidly obese. The interviews revealed clearly that, contrasting with the recalled lack of concern in mid-adolescence,^20^ weight-related concerns and/or desire to lose weight generally increased around the time of school-leaving and most participants described some form of both exercise (formal/informal) and dietary weight-control strategies. These changes may have partly resulted from increasing autonomy (independent/voluntary functioning),^25^ self-determination^26^ or self-esteem^27^ with age: many participants described perceiving postschool transitions as a fresh start and acknowledgement of weight as personal responsibility; most had left the parental home and controlled their own diet and leisure activities.

Differences between ‘slimmers’ and those who had become or remained obese were subtle and hard to detect, even using qualitative methods. A qualitative study of 22 US overweight adolescents, identified via health centre records, found those whose BMI decreased over a 2-year period were more likely to describe ‘transformative experiences’ and family support as well as intense daily exercise.^4^ Other qualitative studies have identified successful weight loss maintenance strategies including dietary change, ‘overwhelmingly increased’ exercise and rigorous self-correction after going ‘off course’ among US 14–20 years with sustained weight loss,^28^ and a ‘healthy obsession’ with monitoring food, activity and weight among eight formerly obese US adolescents who had attended an immersion treatment.^29^ A qualitative study of 20 overweight Taiwanese nursing students highlighted ‘the struggle’, of continuing to practise a new lifestyle and so reducing/maintaining bodyweight.^30^ These findings are consistent with suggestions in our data of lifestyle changes becoming habitual and/or part of identity among ‘slimmers’, and of their appearing more likely to self-monitor diet and PA.

Few participants described receiving professional support and, although numbers are small, diet clubs seemed to have been used most by ‘gainers’. In contrast, ‘slimmers’ had achieved weight loss, without support, sometimes fairly easily. A previous qualitative study of obese Australian adults similarly found that few received long-term professional guidance or support as adolescents.^31^ Although important for adolescent weight loss,^4^ ^28^ it has been suggested that exercise is less acceptable as a weight-loss solution because it is perceived as harder,^31^ yet in this study, slimmers commonly used and sustained exercise as a method of weight-control and did not generally describe it as hard.

Our analysis highlights complex relationships between postschool transitions and weight-control behaviours. University/college, work and independent living were each described as facilitating weight losses by some and increases by others. Analysis of US longitudinal youth survey data has identified subgroups with distinctive patterns of weight-gain risk at different periods from middle-school to work/family formation.^32^ Other studies have found evidence of declines in PA, increases in alcohol consumption and poor nutrition at University^33–35^ ^36^and in young adulthood,^37^ ^38^ but these life-stages have not previously been described before as promoting weight loss. Relationships have also been found between obesity and work conditions including long hours, but again not weight loss.^39^

The main strength of this paper is its objective categorisation of participants as ‘slimmer’, ‘gainer’, etc, based on (measured) BMI at several points throughout adolescence. The threshold used in childhood (95th centile) is not a stringent definition of childhood obesity, though widely used for public health analyses.^40^ When compared with the more stringent clinical definition of obesity,^40^ the 98th centile (Z score 2, equivalent to BMI of about 30 at age 20), nine of the participants were only overweight as adolescents, but it is of note that five of these went on to be obese as adults. Several not categorised as ‘slimmers’ or ‘relapsers’ also mentioned weight loss, not detected by our measurement schedule. Gaps and possible weight changes between measurements, and the sometimes vague nature of participants' recollections mean that precise chronological mapping of these against weight changes is impossible. As the original study did not set out to specifically identify some of the themes highlighted here, particularly professional support, identity and vigilance, we cannot know if other participants might have discussed these issues had the interview included them. The fact they emerged spontaneously is a strength, but because they were not a consistent focus of the study, conclusions on differences between the BMI trajectory groups must remain tentative. However, future research on late adolescent/young adult weight-related concerns, behaviours and experiences could explore these issues more explicitly. Another limitation of all interview data is that participants might have been providing acceptable ‘public’ accounts to a public health researcher^41^ about a stigmatised issue.^42^ ^43^

In conclusion, this exploratory paper adds insights on experiences of obesity and weight loss during a rarely studied life-stage when research participants are hard to access. In contrast to their recollections of adolescence, as young adults even the heaviest participants tended to show contemplation or preparation for weight-loss action.^12^ ^13^ Although there were few really distinctive differences between those who successfully lost weight and those who became ever more obese, their accounts suggest the importance of social context and highlight potential health-change opportunities during the transition to adulthood. This could be a key life-stage for interventions, which should include workplace and educational^44^ settings.
