# Supplementary material for: ‘Now I care’: a qualitative study of how overweight adolescents managed their weight in the transition to adulthood
Source: BMJ Open. 2016 Nov 2;6(11):e010774. doi: 10.1136/bmjopen-2015-010774 (PMC5128912; doi:10.1136/bmjopen-2015-010774)
Supplement: supplementary file [file bmjopen-2015-010774supp1.pdf]

Interview topic guide

Introduction

Firstly I'd like to thank you for taking the time to speak with me today. As the information sheets says, I am interested in how teenage years affect adult life. There are a number of areas of teenage life that I am interested in such as health, friends, family and school. This interview will be broken down into a couple of main areas

Firstly as an icebreaker and also to get an understanding of your perceptions of health, I'm going to give you some picture cards to look at and describe to me.

Secondly I'd like to talk about what you remember about being a teenager: school, friends, family, etc.

Thirdly I'll ask you to tell me about how much you think you have changed since you were a teenager.

While we are discussing different aspects of your teenage life, I am going to provide this timeline which as we go along I might ask you to mark on it when you remember certain things to have happened. Don't worry too much about it, it's just to help you remember more easily, and helps me understand more about your teenage life.

Section 1: Perceptions of health

Picture task (examples below – shown in pairs):

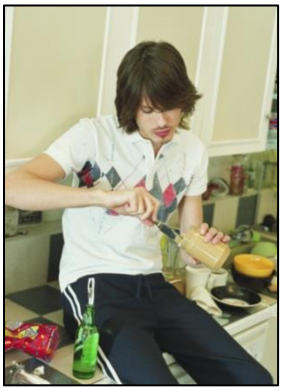
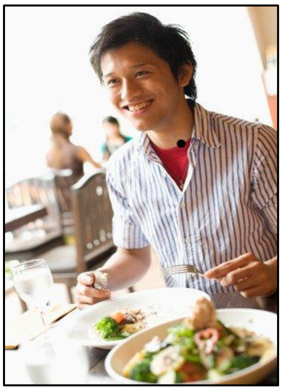
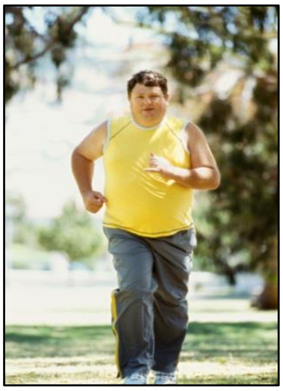
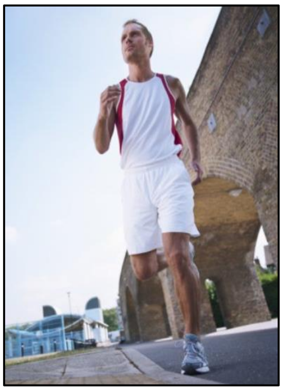
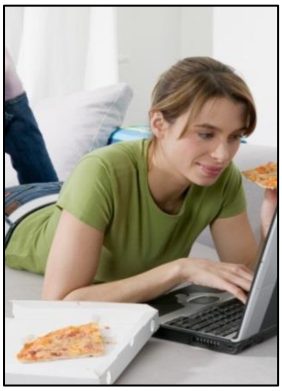
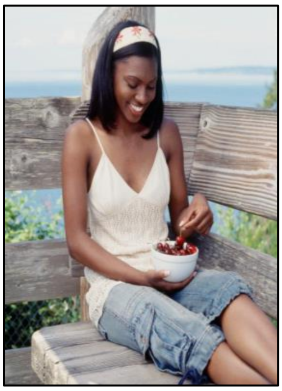
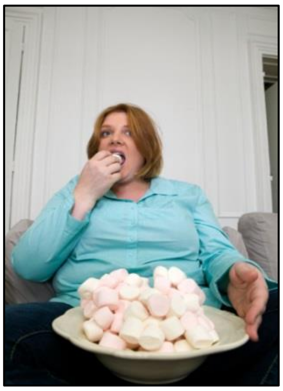
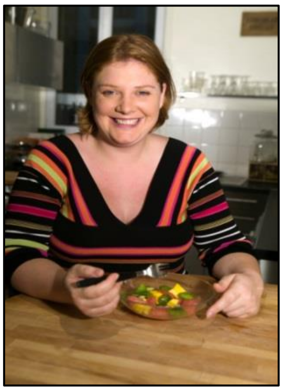

(Images sourced free from Getty Stock Images.)

- I'm going to show you some pictures, and I would like you to begin by sorting the cards into the categories **healthy** and **unhealthy** as best you can…
- Can you now tell me your reasons for putting the pictures into these categories? What do you think healthy and unhealthy means?

  Prompt for
    - Are the people un/healthy
    - Diet
    - Bodies
    - Activities
- Can you now do the same again but sort them into whether you think they are **happy** or **unhappy** in their lives?
- Who do you think feels good about themselves?
- Tell me in as much detail which of the pictures best represents you as a teenager. It might be more than one picture and that's ok but give me your reasons for your choices
- What do you think of these pictures? Do you think that the gender of a person makes a difference to your opinion of their health and happiness?
- Was health an issue for you that you remember? Prompt for?
    - Diet
    - Weight
    - Smoking
    - Drinking
    - Medical conditions

Section 2: Perceptions of being a teenager

I would like to begin this section by asking you to talk with me about what you remember it to have been like to be a teenager;
- Tell me in as much detail as you can, what it was like being a teenager
- Looking back, how would you describe yourself as a teenager?

I'd like to go on and ask you some more specific questions about being a teenager but as it's a fairly long period of time, I am going to provide this timeline which as we go along I might ask you to mark on it when you remember certain things to have happened. Don't worry too much about it, it's just to help you remember more easily, and helps me understand your teenage life.

Teenage life
- What sort of things did you get up to when a teenager? Prompt for
    - Hobbies & interests
    - Jobs
    - Social or anti-social behaviour i.e. drugs, smoking, drinking, etc
- Was there anything you wished you could have done as a teenager but didn't for some reason
    - What was this
    - Why didn't you participate in this?

Significant people
- Could you tell me who the people are that stick in your mind the most from being a teenager and for what reasons? Prompt for;
    - Friends or peers
    - Family – parents, siblings, others
    - Teachers

- Which of the people you remember from being at school do you feel was most like you and which were the most different to you? Why? Prompt for;
    - Physical differences
    - Social differences
    - Emotional differences
- Was there anyone you knew that you really wished you could have been like? Prompt for why?
- Which of these people still impact on or adult life do you think, and for what reasons?

Teenage Concerns

- Do you remember having any concerns while a teenager? Prompt for;
    - School
    - Social – friends, peers, family
    - Health related

Section 3: Change after school

- As all people change during the teenage years, I would like for you to tell me how you changed. Prompt for:
    - Interests
    - Friends
    - Fashion
- One of the things that you were asked to do in a past questionnaire that you may remember, was complete a scale of how you think your body size and shape changed between different stages of being a teenager. I don't know what you completed back then, so I would like for you to tell me again how you think you changed. We can also mark it on the timeline if that helps.
