# Supplementary material for: ‘Now I care’: a qualitative study of how overweight adolescents managed their weight in the transition to adulthood
Source: BMJ Open. 2016 Nov 2;6(11):e010774. doi: 10.1136/bmjopen-2015-010774 (PMC5128912; doi:10.1136/bmjopen-2015-010774)
Supplement: supplementary file [file bmjopen-2015-010774supp2.pdf]

Supplementary File 2: individual BMI Z-score trajectories

'Slimmers' – now normal BMI

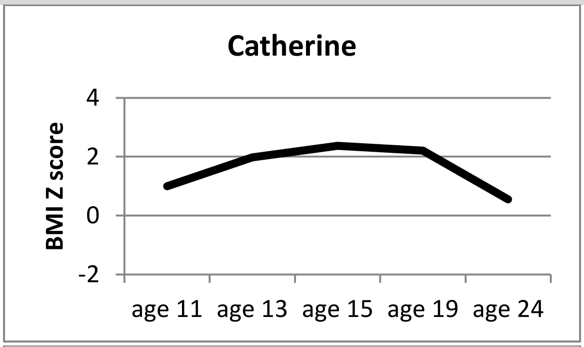

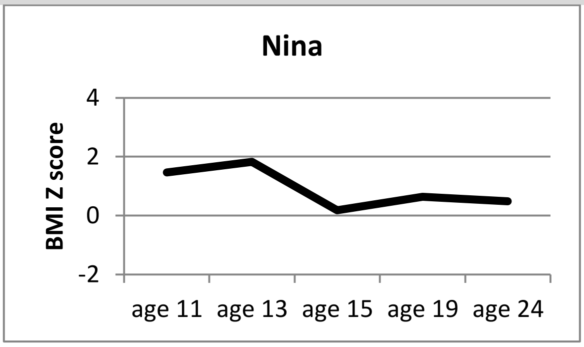

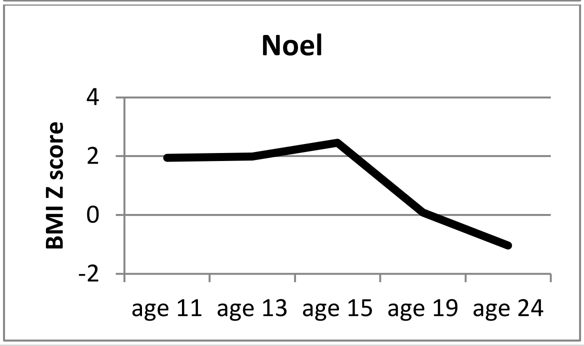

'Slimmers' – now overweight BMI

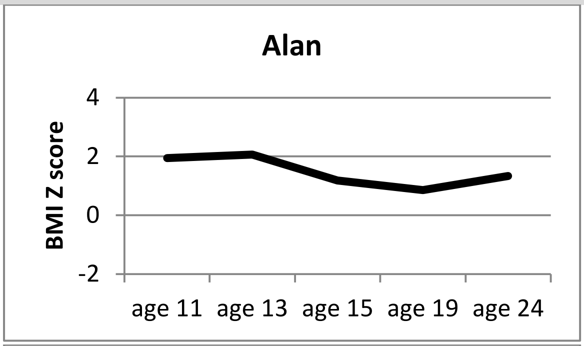

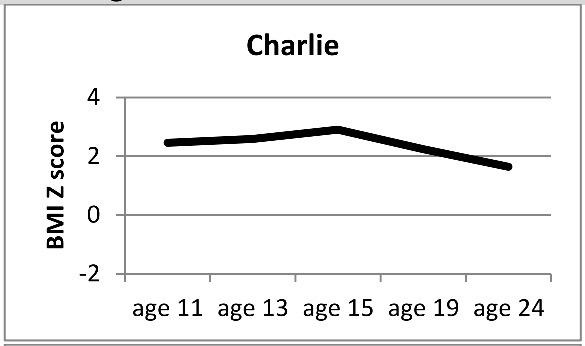

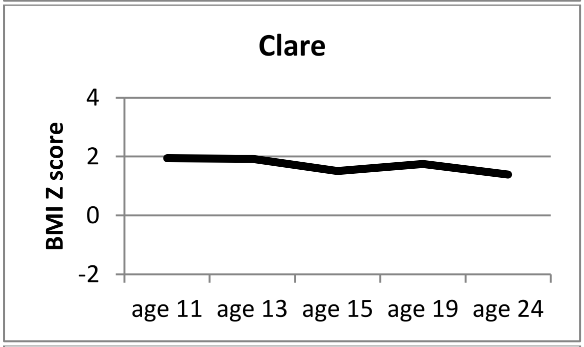

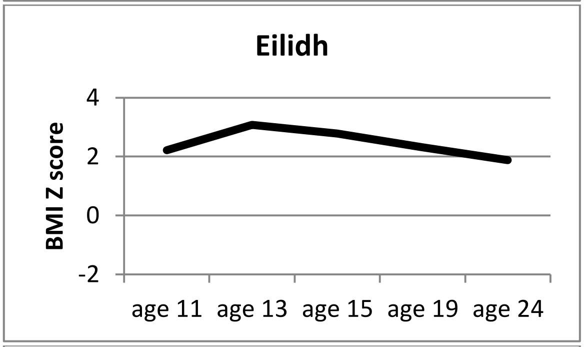

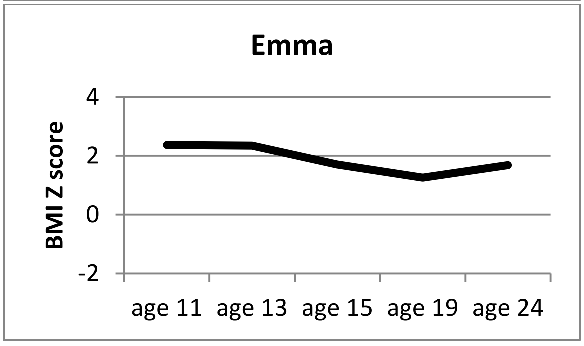

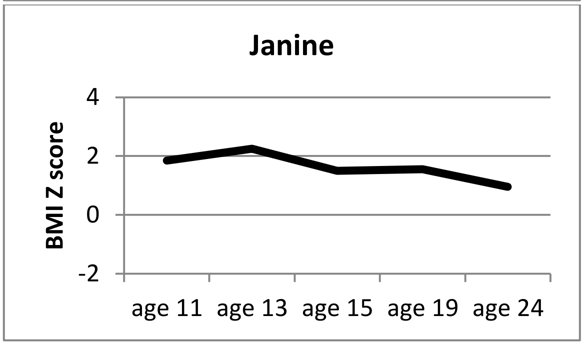

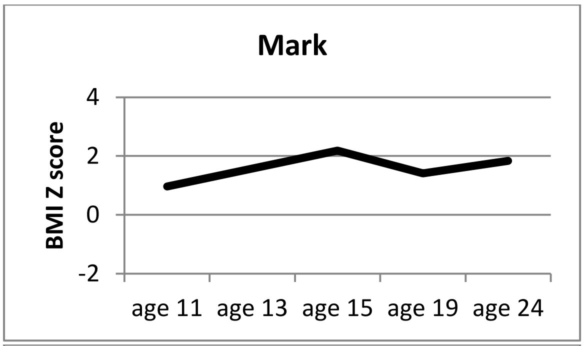

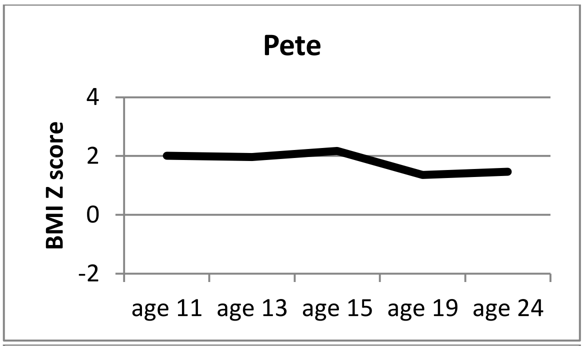

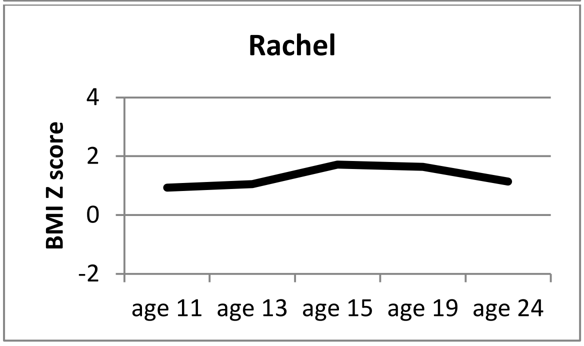

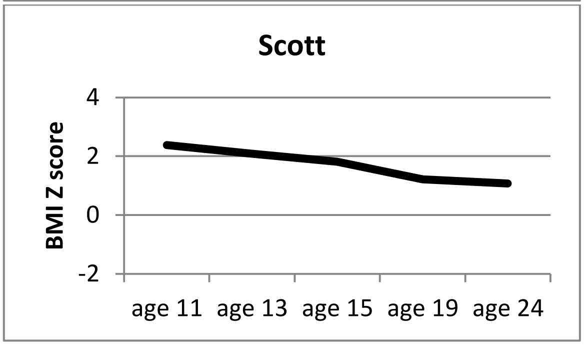

‘Relapsers’ – now obese

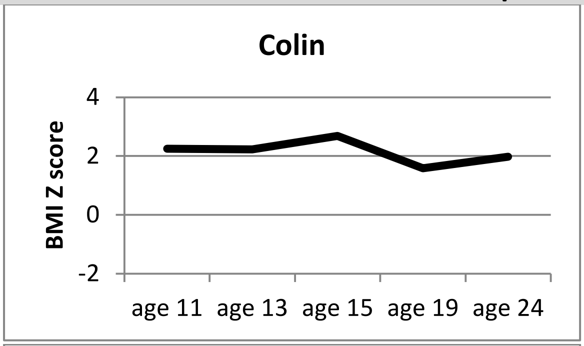

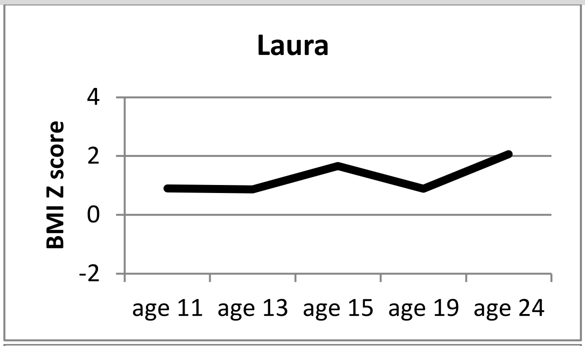

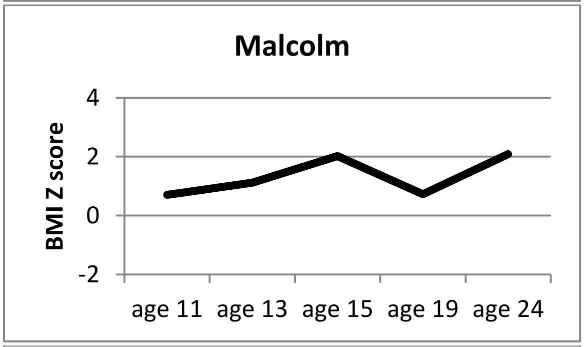

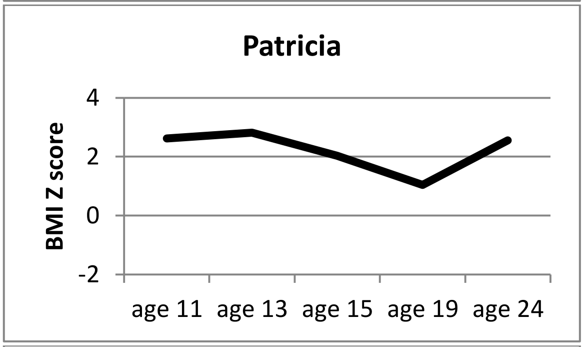

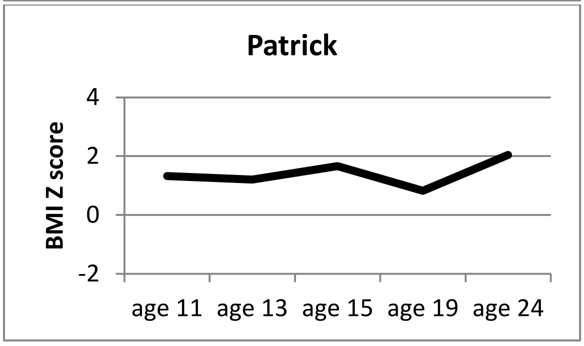

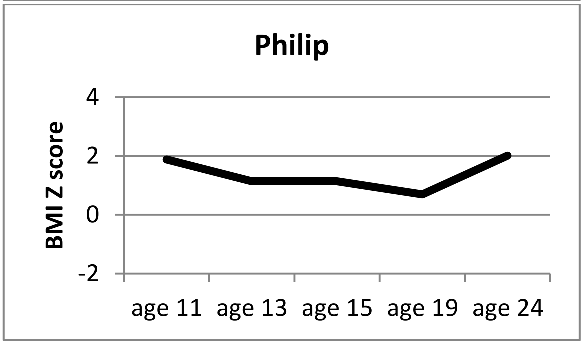

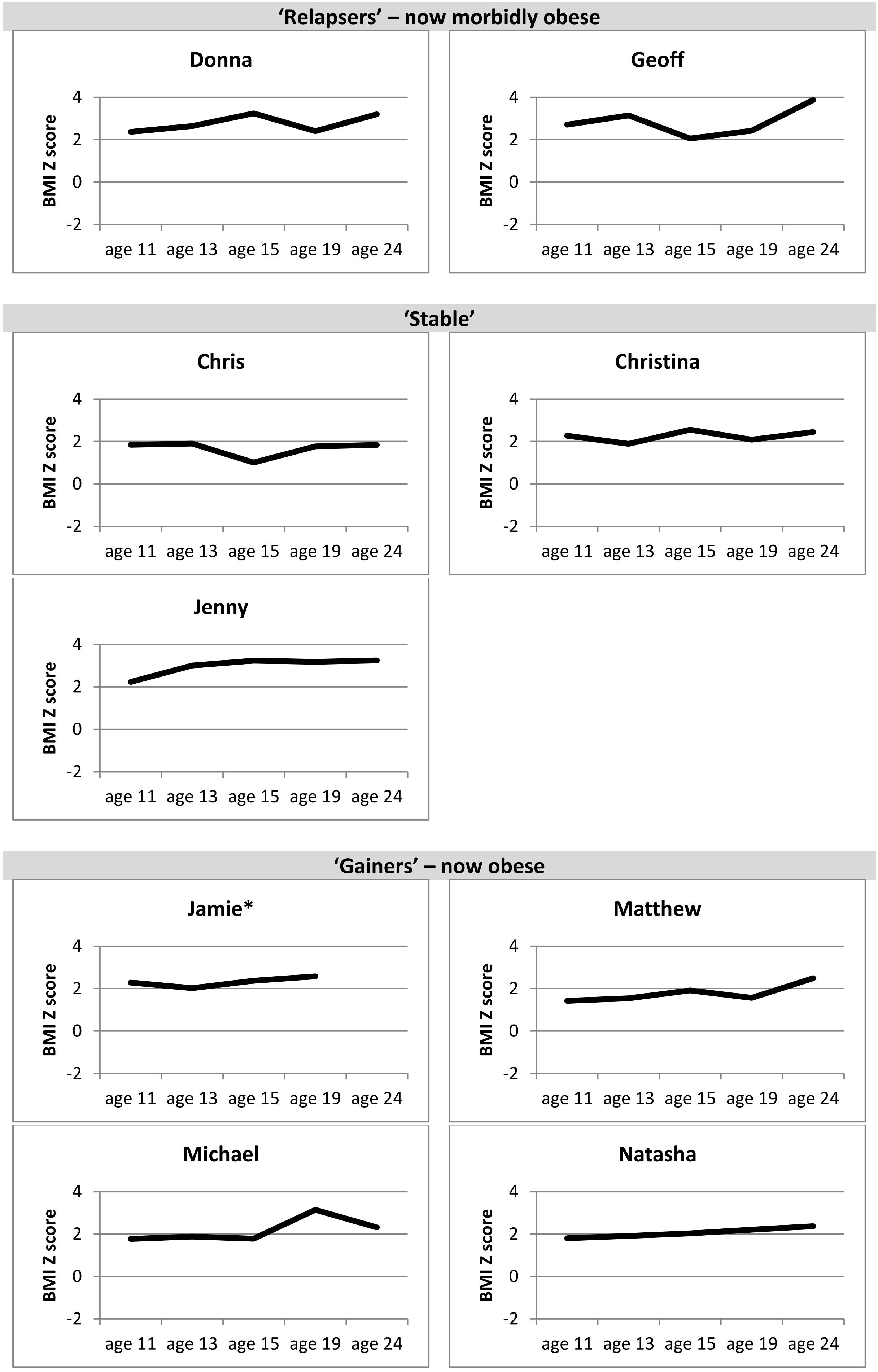

'Relapsers' – now morbidly obese
Donna
Geoff
'Stable'
Chris
Christina
Jenny
'Gainers' – now obese
Jamie*
Matthew
Michael
Natasha
BMI Z score
4
2
0
-2
age 11
age 13
age 15
age 19
age 24

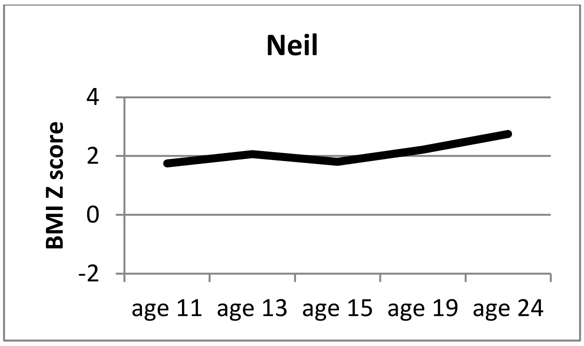

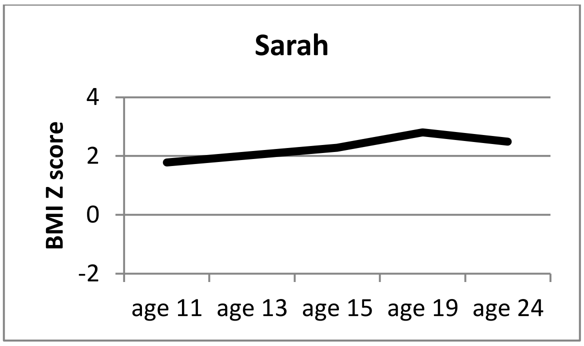

‘Gainers’ – now morbidly obese

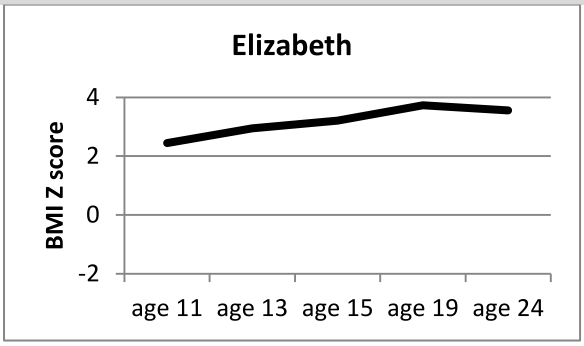

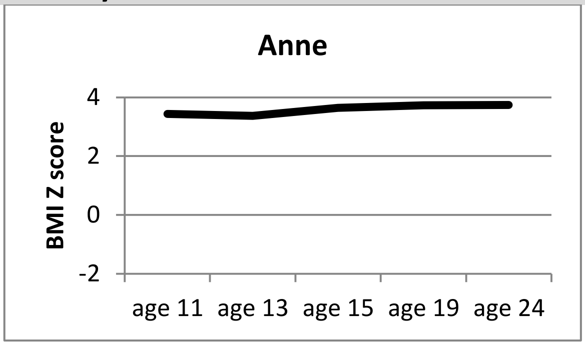

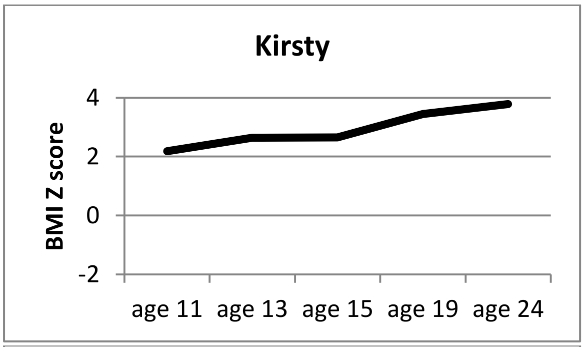

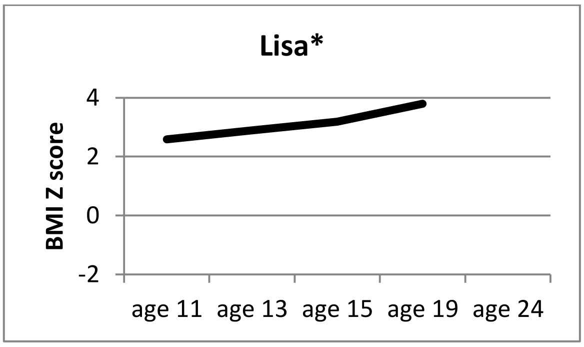

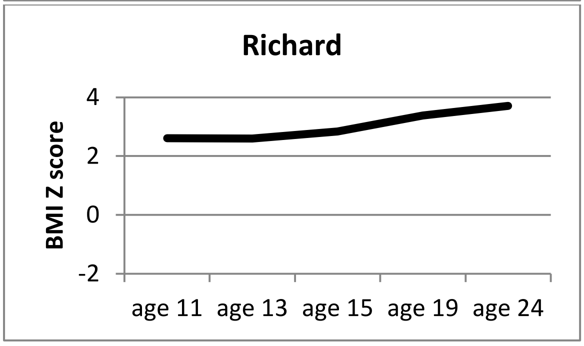

* Jamie and Lisa did not consent to be weighed at age 24
