# Supplementary material for: ‘Now I care’: a qualitative study of how overweight adolescents managed their weight in the transition to adulthood
Source: BMJ Open. 2016 Nov 2;6(11):e010774. doi: 10.1136/bmjopen-2015-010774 (PMC5128912; doi:10.1136/bmjopen-2015-010774)
Supplement: supplementary tables [file bmjopen-2015-010774supp_tables.pdf]

**Supplementary Table 1: Illustrative quotes according to participant 'slimmer', 'relapser', 'stable' and 'gainer' categorisation – weight-related concerns**

| | |
|---|---|
| **SLIMMERS** | |
| Catherine | when I left there to go tae college, that's when I started losing some of it, coz I think, at one point, I was hitting a size sixteen and that, to me, was actually quite traumatic, … So I'm a lot healthier, today, than what I was back then … |
| Nina | I don't know what sparked it off, I think just when you go to high school you realise that you're different from all the other kids …<br><br>But I do notice if my weight goes up or down … So it's still there. But I know it's not the most important thing if you know what I mean. |
| Noel * | |
| Alan | |
| Charlie | There was a definite change I dunno whether it was cos o' me, you know getting to the age o' sorta twenty, twenty one but. Or it was just going to university and – I think it was actually coming o' age that's- which made the cha- big change.<br>[attended gym, lost weight] Now I just- now I jist sorta sustain it. |
| Clare | … but obviously once I'd got though my fifth and sixth [secondary] school year and then got my place at university and things like that, think 'oh dear I'm a bit unhealthy', you know. Don't quite fit into these jeans and things like that.<br><br>.. you know if I was to put on more weight or something like that, I wouldn't- it wouldn't make me unhappy, I would just go out and do something about it. |
| Eilidh | Yeah I don't know I think when I started coming to the end of high school and realised that I was going to Uni, I didn't want to be big, it was like a new kinda fresh start.<br><br>Once I got to a size 16 I just got kinda lazy and went 'well, I'm fine now', do you know. I'd, I would like to lose a wee bit more but I'm quite content the way I am do you know. |
| Emma | |
| Janine | probably from about [secondary school] fourth year onwards, I started watching what I was eating and lost weight so it changed in high school. … it was conscious, because, em, I wanted to lose weight. |
| Mark | it was only until I was in my late teens that I started to think, or I started to be aware of this concept of healthy living, yeah it wasn't something that ever kinda touched me as a, as a fifteen year old boy. |
| Pete | [At age 19-20] I think I just felt that I had to do something about it. I just felt uncomfortable. …. I felt disappointed in myself that I could be doing more. |
| Rachel | I did diet, not like … probably not till I was about fifteen, sixteen … I got a bit older, I started being more aware of what I was eating and trying to lose weight.<br><br>… it doesn't bother me the way it used to, like weight… I still, I exercise more now than I used to, I go to the gym a lot and stuff, but I kind of realised as I got older that I was never supposed to be a size six or a size eight, that's just not the way I'm built. |
| Scott | even now I am very aware of my weight and it's something that I try to look after … the lifestyle wasn't so much a big thing about until I turned maybe 18/19 and started doing my degree … |
| **RELAPSERS** | |
| Colin | I would say about sixteen, seventeen is when I totally got to the stage where I disgusted by mysel', because I was getting to the stage where I was going out and you were meeting new friends and stuff like that, and you were kinda, like getting more sociable, and I just hated my appearance.<br><br>Now I know I've put on weight, but it doesnae bother me, not half as much as it would have done when I was younger. |

| | |
|---|---|
| Laura | |
| Malcolm | I still left school thinking, "nah I don't care about dieting", again "if I eat I'm just gonna burn it off, quicker than anyone else" and then that kinda stopped and I was like that "oh wait a minute, need to try and do something". Didn't really get into that. And now it's just a case of, you can- you can exercise as much as possible, if you keep on eating it's just not gonna be worth it.<br><br>Well want to lose more weight but … just need time. Don't think I'm at risk. |
| Patricia | so I know why I was unhealthy in my teens but I have tried as I've become more responsible and conscious I've been trying to lose weight and have been trying to be as healthy as I can …<br><br>And then now I don't feel as bad because 16, you know some of my clothes are 18s depending on what I wear it, it's fine though cos I can find things nearly everywhere I go. It doesn't matter what shop I seem to go in, I can find things. |
| Patrick | [comparison with earlier] I was quite into running and doing half marathons and that later on, probably aboot 17, 16, 17 maybe. So I done a few of them and I thought that was the start of me being a kinda new me, a good fit kinda outgoing me.<br><br>I would say I'm a bit less determined to kinda change my lifestyle and my fitness and my, my physical appearance and that. But that's, I'd say that's more to do with because I've got more on, I get up earlier, I come home later I've not really got time I'm kinda tired at night and that and I'm, it's not because I don't want to it's just more that it's an effort to I suppose. It's easier to sit in the house isn't it? But hopefully that'll change but.. |
| Philip | Eh more recently em, I suppose I've, I've occasionally, not always but occasionally made a concerted effort to choose healthier foods and things like that. Which hasn't- which was never really a concern until I moved away from home and that- then aye, it's since I started like buying my own shopping and doing ma own cooking. |
| Donna | [re recent weight-loss attempts] Just because I've kinda got fed up with it. It's reached a point where I'm like I don't want it any more so I've just kinda got to get rid of it. |
| Geoff | And I wouldny say I wis unhappy in ma size or anything but I'm no ower the moon aboot it obviously but it doesny get me doon or anything. This is whit I'm are, this is who am are. But as I say if I could get healthier, or fitter rather, then I would aye. But I wouldny be overly concerned aboot it. |
| **STABLE** | |
| Chris | I think it probably started about, like to actually bother me, when I was about fourteen, fifteen sort of time, I'd say. |
| Christina | … it was mair so, see when I was younger, I was mair so that I wanted wee-er boobs so that I could fit into wee-er things. Because o' the teenage stuff [clothes] didny go up tae my size, do you know what I mean?<br><br>I just kinda tapered oot kinda thing. Em but I don't think lassies are ever happy. …But I'm quite vain, even though I'm big, I think I'm shit hot, do you know what I mean? … I am quite vain, even though there's things that I would like tae change, but I'm no gonna bust a but tae change them, do you know what I mean? |
| Jenny | Like yoga or do different sort of things to try … Never so much that oh like you had to go to all these classes to get healthy. As long as I don't feel like crap I'm not too bothered like. |
| **GAINERS** | |
| Jamie | At the end of second [university] year I was oh really big and then I actually went on a really big sort of summer dieting phase at that point. And I dropped a lot of weight that summer.<br><br>I mean my weight has actually been quite steady. You know it's, it's too high but I haven't put on weight for a while |
| Matthew | As I became fifteen, sixteen I was unhappy as how I was. So I wanted to change that and- and just through that I became more confident and a lot happier. |

| | |
|---|---|
| | … eighteen, nineteen where I was very comfortable as I was and none of it really mattered and I could drink what I wanted and ate what I wanted and play football when I wanted and it was just sort of- sorta plateaued as it were maybe |
| Michael | It's only been ower the past year or two that I've knuckled doon and starting to get it off. …I've never tried to do anything about [the weight] … until I was old enough to realise. |
| Natasha | I think like in fifth and sixth [secondary school] year, like weight went and got an issue for me, you know, like I wanted to lose weight … kind of wanted to just look better, like slightly thinner, thinner.. |
| Neil | I love my grub. It's just the way I am Eh, I think I'm always gonna be a big lad …. Em, I could lose, eh, weight, eh nae problem, but it's more a case of, I don't find it a real necessity … |
| Sarah | I am now finally on a diet for the first time properly in my life, so I've joined Weight Watchers a couple of months ago so I've now lost just over a stone I think I'm going back tomorrow to get weighed again, but so I'm finally trying to do something about it cos it bothers me. I don't think there's much else that's changed really I don't know, I'm far more mature than I used to be. |
| Anne | I've always been overweight from a child since I was em a toddler … but when you're younger you think, I don't care … But as I lear- as I got older I realised that I had to do something, I had to stick to what I was told otherwise it was just gonna get out of control kinda thing so. … obviously I'm more, I'm more health conscious than what I was as a teenager, I think probably most people do when they grow up. |
| Elizabeth | So I have changed since I've been a, teenager because I watch what I'm eating and, I only have a bit of chocolate maybe once a week and, I don't go out and just pure indulge because I want to. |
| Kirsty | when I was a teenager I was never bothered aboot my weight, it just never bothered me, but noo it is, I've started Slimming World and started going to the gym and things so, you know like I'm ready to take that step to lose weight, but when I was younger, it didnae bother me, I don't think I really had any concept of being big you know. |
| Lisa | [as teenager] I was always trying really hard to get fit an' lose weight and whatever. |
| Richard | By the time I got to about fourteen, fifteen, that's when, obviously, you start looking at yourself a wee bit mair … when you're in high school and that, you start daen things like swimming and everything … but I was getting bigger – you've got a wee flabby belly and that, and you're like 'I don't really wanna go swimming noo'.<br><br>When I left high school, I started making money and got lazy and didnae go oot as much, like, exercise-wise, started drinking and then just ordered take-aways. … just sit aboot and have a laugh. |

**Supplementary Table 2: Illustrative quotes according to participant 'slimmer', 'relapser', 'stable' and 'gainer' categorisation – exercise**

| SLIMMERS | |
|---|---|
| Catherine | Normally, what me and my wee boy do is we go walks down the, we've got the big woods down there, and we've got the waterfalls and we've got the canal with the swans and that in it … Knocks him out for his bed, and gies me a bit of, a bit of activity as well, wae burning some calories off as well, so he loves that, and half the time I need to chase after him, so I'm burning more than what I think. |
| Nina | Yeah exercise really helps and that's something I have neglected just with where I live walking into [named] town centre and whatever it's not, it's not the best but I have been doing it I've been going for walks, going for the odd swim, whatever but I should be doing more cos I do notice it in me that it, that it helps. |
| Noel * | 'd say I probably dae a lot mair exercise noo, you know than I maybe did when I was… like when I just first left school you know. |
| Alan | [Notes only – Needed to improve fitness for admission to RAF so began running and cycling – didn't lose weight but increased muscle mass and lost fat.] |
| Charlie | And then I dunno if it was coming o' age or, it was just that I started going to University, was it four years ago now and eh, started going to the gym. Then that's the reason- well that's the only reason why I've lost- lost- I lost like four stone. Just going to the gym. And it's not something I was going to- it's one o' those things that I- I- it was about a year and a half I lost the weight in about a year and a half. And it's- it wasn't even difficult for me. And just- just one day I just- do every- I started going to the gym go- and I wasn't struggling, doing it I was- just- a total change in lifestyle. … before I know it I wis going every- I wis- at that point when I wis losing the weight a wis two or three times a week, like- like really, getting really involved with it at that point was losing weight. Now I just- now I jist sorta sustain it. |
| Clare | … cos [university] had a gym in it, I used to use that all the time …Not all the time but, I used it throughout my Uni life. … you had you know a free period here, free period there and things like that, you had your gym- I used to have my gym stuff with me, and just carry it with me and usually just go to wee, half an hour or do a wee class, aerobic class or something like that, and just do that. Probably just got me into a habit, now I'm kinda used to it. It's not like I'm doing it because I- 'oh God I need to go and exercise', I just- kindov habit. … I mean I don't think I have to go to the gym or do this, to exercise I would just do, walking, jogging, whatever. Walk to the supermarket and back to get some bread and milk. |
| Eilidh | I cycle a lot now. Me and my boyfriend have both got bikes. And I swim a lot as well. Again, I tried going to the gym last year when it was bad weather for cycling, but I lasted a few months and that was it. I get fed up with that but, doing things like if I'm cycling if I know that I'm cycling to a place, like going into town or something or, like I still really love doing that. So that way I'm more fit. |
| Emma | I was nineteen when I joined, and I'd gone, I'd gone down to ten and a half stone, and they told me I still had to lose more weight [for police force] … and you do all your training up there, and you're out running, like, constant … the evening, you're still going, you're going to the gym or you're going swimming or whatever, d'you know what I mean? So I mean, it is the most active I think I have ever been in my, in my life. Em, you're just constantly getting, and that's the reason why I had obviously kind of came down to like a size ten. |
| Janine | … most of the jobs that I was doing was dancing and singing so I was in the gym all the time and I was dancing in shows every night |
| Mark | I don't remember the moment of making the decision, but I do remember coming home from school and getting changed and going to the gym and that was, that was very… I don't know, thinking back on it now, that… it was a bit of a departure from the way life was for me before then because I would come home and either go out and play football or do my homework or just sit and watch telly, and it wasn't like I did anything else in particular anywhere else, but I started doing that<br>I joined the sports union, so I joined some sports clubs, so I got my gym memberships so I started going there. … it became part of my life and it has remained so to this day. |
| Pete | Yeah it was just after that so I joined the gym with him and he gave me a lot of encouragement so that was good. So I felt a lot better within myself but even though I don't think I lost an incredible lot amount of weight but you know I still felt better for having done it and taken positive steps. So yeah. … I think by the time I left school I was exercising a lot more. And you know taking more care of what I was doing so I don't think very much, I don't think it's changed very much since then so I think I'm a wee bit more mindful of what I have to, you know try, try to do in terms of eating habits and things. |
| Rachel | …. when I started exercising I felt more awake as well, I had more energy, so that made it more enjoyable as well, especially seeing the benefits from it. … I don't really do |

| | |
|---|---|
| | or have any other hobbies as such. Between uni, work, go to the gym and then… that's you just socialising, I don't have the time to do anything else. …Now I got to the gym a lot and take- I do different classes at the gym. |
| Scott | The lifestyle wasn't so much a big thing about until I turned maybe 18/19 and started doing my degree then I started learning how to use a gym properly and what sort of exercise that I can do and just I'm now very aware of cos I'm working in nutrition what it is I actually take in and what it is I actually expend …Now I've got a little bit of a phase where it's eat shite and train hard |
| **RELAPSERS** | |
| Colin | … at one stage, because I kinda ballooned out to quite heavy state. I kinda went on a fitness freak stage, and I just constantly, I wasn't running about and stuff – my exercise would content wae me being in my room, wae my music on really loud and just dancing about, coz I love dancing, and I would still eat whatever I wanted to eat, but I would go in – if I ate something that was overly, too fatty then I would, like dance for like three hours in my room, non-stop. |
| Laura | I've got an exercise trampoline in the house, so I tend to try and use that a couple of times a week – not that I always do, coz, I will confess, em, you know, there's just those weeks where you just can't be bothered – but I actually love my wee trampoline. IPod on, trampoline, let's go. |
| Malcolm | There's not been any exercise really, not much. Apart from like always did kickboxing things like that. But mainly it's just all food. |
| Patricia | When I was in my very late teens, like seventeen, eighteen, I lost a drastic amount of weight and I got to like a size eight to a ten, and I was exercising an awful lot.<br><br>I feel like exercise and stuff's a lot easier now because it's not like, like I say my mum would give things up quite, quite easily, whereas [boyfriend] and I will go and it's just accepted you know these are our days we go to the gym and that's it. |
| Patrick | Later on, after I left school or I was quite into swimming. I worked up at the sport centre as a lifeguard so I was really into swimming at that time and a bit of running as well. … [Football] That's taken a back seat. I'm quite into I suppose when you're a teenager you can't drive so I'm into cars now, I'm really into like my car and that kinda thing. That's my main interest, I'm trying to get back into swimming, I'm trying to make an effort to go swimming a bit more now. And when the good weather comes in I'm going to start running again. So that's not changed as much but it's just circumstances wi' that, that I've no got the time just now but I'm gonna make the time for it. |
| Philip | I tried to do exercise but not, not specifically training. Em, I still play rugby, not- not that I- not that I go to training sessions or team practices or anything like that. |
| Donna | Yeah aye exercise as well because I've, I've been trying to keep at least the healthy level likes of being as active as I can, not driving and walking wherever I need to and taking the dog for a walk when I'm home type thing. |
| Geoff | When I left school I went to I done, I done boxing, fitba, I went to the gym. … I wis I say I wis playing aw the sports. So if I could eat that but I, I wisny putting on any weight cos I wis going to the gym, playing fitba and that. I don't play a lot o' fitba noo right enough. I'd like tae but it's getting the time and the people tae play it. Cos a lot o' ma pals aw work like different shifts but I, I don't think I could go back to the gym noo. |
| **STABLE** | |
| Chris | I mean, you can see I've obviously got the treadmill here, so I'm still you know, quite, quite into keeping fit and stuff, so… but I didn't really you know, I never did anything particularly excessive. I never did anything too… you know, tried… sort of stuck to anything very long I don't think when I was, when I was younger, so I guess that's probably why nothing ever worked. |
| Christina | So I wouldny go tae a gym or nothin' like that that's how I got her. I take [her dog] walkin'. Just go tae the braes wi' her and that's aboot it. … So you walk up there and walk back. That's, that's a fair, it's a fair walk do you know what I mean? Or even walkin' roon, there's three parks there and you go through the forest and through tae the other, do you know what I mean? I do that three, four times a day. |
| Jenny | The job I'm doing now … it's like eight hours of power walking, pushing this massive trolley. And I've been doing that for like five months now and I've noticed a difference, you feel a lot better doing that. |
| **GAINERS** | |
| Jamie | In between the sort of May and October you could say of that point in second [university] year, I started to exercise, I started lifting weights. And went on a sort of you would say diet and I lost nearly two stone, maybe a bit even more. Cos I put on a lot of muscle mass as well. … I started to do more exercise and yeah I lost a lot of |

| | |
|---|---|
| | weight. And I was quite, and then that sort of continued through the end of my third year. The rate of loss sort of, I stopped at being a massive, but I did get even lighter towards the end of third year and in the end of my year is probably the best shape I was in …, at the end of third year it was like fourth year kicked in and academically I was a bit behind. So I really needed to dig, to dig in. The gym work kinda dropped off a little bit. |
| Matthew | [around age 16] And it was only when I kind of realised more if I worked out, that I would change so, you know as well as with running and playing football I started going to the gym I noticed the difference in my physical appearance and so, like whereas a lot of my friends are skinny and fit, I was a bit heavier but more muscular …<br>I've put on a lot of weight certainly since, since my later teenage years. Like you know like em, cos I don't play football at all any more, or I don't run, go to the gy- I don't do- it's very rare you'll see me going doing something now. |
| Michael | … used to hit the gym, and I bought myself, like the multi-gyms and aw that for my room, but because we moved here, then there's nae room for it, I got rid of it aw, but I still go to the gym noo and again when I've got time, fae work. Aye, I'm still cerrying on wae that. |
| Natasha | [around age 16] I was dancing a lot more – cause that point we were in, like, kind of doing a lot more dancing. The dance shows and stuff.<br>[currently] I don't dance, but then I kind of, like, I joined the gym and I kind of, I do that kinda thing … |
| Neil | I got addicted to, eh, to building myself up. I was going to the gym, eh, wae my [named] uncle every, eh, two or three times a week. In between that, I woulda been, probably playing football or just chilling out in the house, but, eh, physically and psychologically, so much healthier… so basically fae sixteen tae, up to about twenty, I was going, really common, then work commitments and money and relationships [meant he stopped] … |
| Sarah | [No discussion of exercise, except not enjoyed at school. Currently attending commercial slimming club with diet plan] It's been a, quite hard cos I've been on holiday a couple of times this year and it's quite hard to stick to it when you're away so \i just try and do quite a lot of walking when I'm away … |
| Anne | I used to go to the gym on a Monday but it's shut now, the gym that I go to, it's not opened anymore. Em, for refurbishment. But like, I've got like exercise DVDs now that I'll do in the house. |
| Elizabeth | Like, I don't like- you hear loads o' people going on about 'oh I love going to the gym and, I like doing this and I like doing that' and I just (laughing) go 'oh no I can't', so. I like things that are in the water like I like aqua aerobics and things like that I like things that involve water because, I don't know I just think that when I first started I thought it hid me, more than anything else, know that way 'cos you're dead self-conscious and you think, if I go in the water I'm just gonna hide in the water so, that was it I just hid in the water and I swam back and forth and- kept myself happy. … Uhu because I found that, eh, I'm going swimming a lot more and I'm goin to the aqua-fit and I'm doing, all these things that when I was a teenager, I'd never even dream a doing. |
| Kirsty | I've been daen yoga for six weeks as well, and then I joined, just joined the gym last week, so I'm just getting into that the noo |
| Lisa | I don't mind doing certain [physically active] things, but on my own terms and what I like to do … Just running for running's sake I just don't understand and never have … I don't mind as some part of some kind of a game, I see a point to it, I just don't see a point to running a hundred metres as fast as you can. |
| Richard | So it's mostly, you know, the gym or five-a-sides after high school. That was aboot, that was aboot my exercise, but I was daeing it every day when I was in college … and the guy was daeing a session, you had to dae everything he telt you, so you'd still be running aboot for nearly two hours at a time…. so I mean, during college I was fit and healthy and I was playing football every day and going to the gym .. and then when I broke my ankle I just couldn't play anymore. …I started getting knee pain … and now I've got arthritis in my left ankle and they didn't say it was arthritis in my knees, but it was. |

**Supplementary Table 3: Illustrative quotes according to participant 'slimmer', 'relapser', 'stable' and 'gainer' categorisation – diet**

| | |
|---|---|
| **SLIMMERS** | |
| Catherine | If you're gonna comfort eat and stuff your face full of grease, aye, you're gonna put weight on, you're gonna get fat – whereas, if you watch what you're doing, not eat things, you can still eat things that are greasy, but as long as you're draining that grease out … So aye, it was losing the weight, it was, it was hard at the start, but see once you get into a routine of knowing what you do, what you can eat, what you can't eat, what you need to keep yourself away fae, it is quite easy. |
| Nina | So around thirteen, fourteen, I stopped eating dramatically. I wouldn't say I was anorexic or anything extreme but I did, I didn't eat enough for someone my age and I did lose it very quickly and I lost a lot of weight about thirteen, fourteen. |
| Noel * | Aboot seventeen I took a post viral disease … everything I was eating I was being sick. Just couldn't haud anythin' doon. And I lost hunners of weight but just dead, dead quick, you know scarily quick. … [Currently]I don't eat anything greasy, well I don't eat like fry ups or anything like that anymore, you know. It just kinda turns my stomach you know, greasy foods. |
| Alan | |
| Charlie | … if you're eating that much you're trying to stop but you're trying to go on a diet mentally that's a really da- extremely difficult thing to do. I've found it's not something it's- it's almost a- I, I consider it's almost impossible to mentally you know, do- commit yourself to doing that. |
| Clare | |
| Eilidh | And so when I was probably when I was about 171/2 I decided just to start healthy eating .. really healthy eating …. And getting salads every day and fruit and I lost the weight pretty quickly. … And I didn't hardly even exercise much do you know and it did kinda come off<br>I'm very, very always watching about not getting bigger, do you know. And I'm as I said, I love cooking and that is a kinda concern for food and calorie amount and this, that and the next thing I think will always be with me probably and I will kind of always have that |
| Emma | I do, like, kind of eat my vegetables and, and kind of, like pastas, like good, fresh pastas, and things like that. I don't eat, like, if there's a tomato based sauce or a cheese based one, or a cream cheese, I'll take the tomato one because I know that it's – d'you know what I mean? I'm aware of, of what I can eat and what I can't eat and, like, kind of what's good and what's bad. I don't over indulge, but I do eat the things I enjoy. But I do still, like I say, when I'm having a bad day, I'll go home and I will gorge, and I know I do it, and that's why I hardly ever buy in biscuits and things like that because I know that I will eat them. |
| Janine | … probably from about fourth year [secondary school] onwards, I started watching what I was eating and lost weight …One summer, I just ate, like, I skipped a meal and I would have lunch and dinner and skip breakfast and seemed to lose half a stone and I felt good that people were, eh, commenting on that, so since then, I always kinda watched what I was eating. |
| Mark | I had to start buying my own food, I was sort of… I was able to say things like … "if I don't buy any biscuits, there won't be biscuits to eat". So yeah, I was able to… and that kinda thing exists to this day. I was able to take control a bit more … it was just a much easier kinda point at which to yeah, take control. |
| Pete | I've you know actually followed a Weight Watcher's diet for a few weeks aye, and I lost, I think I lost about ten pounds in three weeks. So I have, I was sort of mindful again of not falling into that trap again of just replacing things with food. And it's sort of a, I would remember what it was like the time before so I've managed to sort of control my eating habits and not drink so much alcohol and you know so I was doing the same sort of things that I did before. |
| Rachel | … and then I dieted and just started… not so much dieted, it just changed the way I ate, rather than actually following Weight-Watchers or anything like that, and exercise, because I wasn't getting any exercise from really from when I stopped doing PE at school, so… |
| Scott | I always will be worried about, not worried about it but I'll always watch it [weight]. And there's times whereby I'll pick up a biscuit and I'll say no, I don't want it, I'm just eating it for the sake of eating it … |
| **RELAPSERS** | |
| Colin | Even at one point, I did make myself sick at one point [to lose weight], but I stopped doing that. |

| | |
|---|---|
| | … don't know how much damage I've done to my stomach, em, but I, I pay more attention to myself now, as in, like, I will try and eat something a bit more healthier. |
| Laura | maybe in the last couple of years or so, in the sense that, yeah, you go out and do lunches with your friends and this and that, and you think that I could really do with cutting some of that out. You know, weekend fry-ups and stuff like that. Trying to be healthier and, you know, the healthy option, so you know, in, at the time, you're like "Oh yeah, this is really nice", and don't get me wrong, some of the food that I do eat is really nice, some not so much. |
| Malcolm | And then after that you started eating, you started noticing things were different. You were getting a bigger belly, you were getting this and you were like, "oh wait a minute, need to stop doing this because it's- gonna end up really huge". That's what you always thought, "I'm gonny be huge". Need to stop, so you just cut down. You're like, "do I want a packet of crisps, or should it, nah". You start talking like- I've started talking to masel. "Should I have a have a packet of crisps?" "Nah I don't think so". |
| Patricia | I got hypothyroidism so I gained a lot of weight from when I was quite slim … and it didn't matter whether I ate healthy or whether I went to the gym, I still look big and still today … [currently] eat quite a healthy diet, like I wouldn't have those biscuits, I would have the healthy biscuits, but I still don't lose weight. |
| Patrick | [As adolescent] My mum always gave me a good dinner, she's quite good with, good balanced dinner. … I mean I would say it's worse now, because you're kinda looking after yourself more and it's easier just to get takeaway and junk food … |
| Philip | Em and in actual fact I probably wasn't eating as- as well as I should. Or as much as I should. Like I was only getting two meals a day rather than three. And stuff like that. Em, which helped me lose weight quite fast but not in a sustainable kinda way because, em, once I did start eating properly and regularly again, it kinda went up and then it kind of levelled off. |
| Donna | It's been a slow process [reported recent weight loss] because I mean likes of yo-yo dieting I've tried that. These fads that you go on. Never work cos you do take the weight off and in like three weeks and then put it back on. They're hard to stick to but it is all about having a healthy kinda attitude towards it and about doing it slowly but just what I've been doing it's been working, it's been staying off. |
| Geoff | I eat chippies masel' but it's no, I know they're no healthy but it's like food that you canny stop. In moderation it's aw right. Food like that but obviously no aw the time. [post-school] … still eating a lot o' rubbish but I wis a lot more active. … So if I could eat that but I, I wisny putting on any weight cos I was going to the gym, playing fitba and that. |
| **STABLE** | |
| Chris | |
| Christina | I've tried lots and lots aw things. Like you stoap, you stoap, you only eat certain things. I've tried liquid diets, but I only had tried that for like a week and a hauf and couldny cope wi' that. What else? Em. Drinkin' vinegar and stuff like that, do you know what I mean I was a teenager, do you know what I mean?<br>[Currently] I don't really care what I'm eatin'. I dae eat quite healthily but it's my amounts. Do you know what I mean cos I love my food. |
| Jenny | I can just eat really good foods and be really good but it never makes that much of a difference. … last summer I lost like three stone because I didn't like the food when I was away. So it was, I ate hardly anything but it wasn't without, it was, so and I'm addicted to chocolate most of the time so that makes a big difference and when you're in America the chocolate's crap so you don't eat anything |
| **GAINERS** | |
| Jamie | just cut out junk, I cut out a lot of carbs I remember. I ate a lot of protein. And I ate, when I ate carbs I ate them at the right times. I used to eat them in the morning and early afternoon, before I went to the gym, before I started like lifting weights. Yeah it was that what I did I remember doing, I remember saying 'no junk'…<br>You really do need a disciplined and healthy eating plan. You know says the man who had a bag of crisps and a Mars Bar last night, but you know what I mean like it is, it is about, yeah. It is about exercise, diet. That's my personal opinion certainly, but what you eat in terms of your body shape and the amount of weight you carry, diet is everything. Exercise is important, but diet is the primary thing for me. |
| Matthew | I don't actually snack at all any more. Bizarrely I don't snack, it's not snacking. And I don't drink full fat fizzy drinks any more. |
| Michael | I started walking to college, walking back, eating a good breakfast of cereal or a bit of toast. Just having a decent lunch in college, and then just having toast or something at night, and I did notice a big difference – starting to see it coming off and stuck to that, and aye, still sticking tae it. |
| Natasha | [In relation to a reported period of weight loss] I think it's just kind of, I was more watching what I was eating as opposed to just guzzling it down my throat, kind of, and |

| | |
|---|---|
| | snacking as much. |
| Neil | I’ve always been a big eater. I love my grub. Eh, it’s just the way I am. |
| Sarah | I am now finally on a diet for the first time properly in my life … I became a bit of a diet bore for a while. And that’s all I talked about I was like, ‘oh do you know how many points are in that, and do you know how many points are in that, this is so exciting and I’m on a diet don’t you know’ but it’s good cos now everybody knows like at work .. So it’s quite good that everybody knows that I’m on it now cos that means that I stick to it. |
| Anne | [After joining a slimming club] But I noticed that as I lost like a couple of stone stomach, my stomach started to get really bad the acid that’s, … and I’ve been on tablets since I was 21 for my stomach. … they’re trying to figure out if they can fix my stomach, so that I can stick to my diet again. … I’d had to eat things to suit my stomach, rather than suit my diet, it was the way it [weight] started to slowly go back on |
| Elizabeth | I eat plenty of vegetables now. But even before then there was plenty that I ate, healthy wise, that was good for us. Obviously when I went to Weightwatchers kind of a learned that. [Reported recent weight increase] And it’s just because I wasn’t, watching so well what I was eating. Not that I was MacDonald’s every day because that would make me sick. I don’t like it that much. But, em, you know it- it changed and it added … |
| Kirsty | … the [commercial diet club] you can eat certain foods, you can eat basically everything, but, they have green days and red days, so, on green days you have, you can eat as much pasta and potatoes and things wi’ a limit aw meat, and on a red day you can have as much meat as you want with a limit aw pasta and potatoes. …But noo I’m quite organised, I plan ahead and know what I’m havin’ and I’ve always got you know snacks if I need, you know different things, and I eat a lot of fruit noo, so I’ve always got snacks like that rather than chocolate and I’ve started eating breakfasts as well which helps a lot, so I’m no wanting chocolate at 11 o’clock. |
| Lisa | [After stopping slimming club attendance] Therefore I stopped- didn’t stop watching what I was eatin, but stopped recording things and checking things. And all of a sudden it was- not all of a sudden but three years later, things are starting to, get worse again. |
| Richard | Like I says, I mean, when I did lose all the weight, I was eating, I was cooking my own meals and everything. I didn’t have a takeaway for six months, just drinking a wee bit more water, I’d cut down on my juice – I was still drinking two litres a day, but I cut it down and it was diet. |

**Supplementary Table 4: Illustrative quotes according to participant 'slimmer', 'relapser', 'stable' and 'gainer' categorisation – professional support**

| | |
|---|---|
| **SLIMMERS** | |
| Catherine | |
| Nina | |
| Noel * | |
| Alan | |
| Charlie | |
| Clare | |
| Eilidh | |
| Emma | |
| Janine | |
| Mark | |
| Pete | I went to you know like my GP a couple of times to try and get advice on how to, you know what I should do. … [advised] just to try and control portions and try to, to count, you know not count calories but be mindful of what the intake was and perhaps to, to exercise regularly you know with, either with friends or you know try and get support you know. So that did help a lot. That did help. |
| Rachel | not so much dieted, it just changed the way I ate, rather than actually following Weight-Watchers or anything like that |
| Scott | |
| **RELAPSERS** | |
| Colin | |
| Laura | |
| Malcolm | |
| Patricia | I was referred to the Council's weight-management service by my doctor, and I went and never lost any weight there, and because I never lost any weight, they just never got back in contact. And my doctor I feel because she's so big, when I go and I say "I would really, really like to lose weight and I'll, I can show you a food diary of what I've been eating, I can show you my exercise, I can show you how much water I've been drinking", my doctor will go, "och it's ok you don't need to lose weight". And she, she sit, you know and I don't like to be rude to people and I mean I actually, to get on the weight management course, my boyfriend had to come with me and actually say "look, she wants to lose weight and that's it". You know and you know and basically then my doctor took note of him because I feel he was far more forceful than I was. |
| Patrick | |
| Philip | |
| Donna | |
| Geoff | |
| **STABLE** | |
| Chris | |
| Christina | So I know I'm no obese, doctor took great pleasure in tellin' me that I wasn't. |
| Jenny | |
| **GAINERS** | |

| | |
|---|---|
| Jamie | |
| Matthew | |
| Michael | it was the doctor told me that my blood pressure was abnormal and my heart rate wasnae right – she says if I keep cerry on the way I was, I was gonna have a heart attack by the time I was thirty-five, and that put the shitters right up me. … I think I went wae the earache or something, and she just, she says “You’ve no been here for a while, is it awright if I dae this and that?” I said “Aye.” And that’s when she telt us, and so I scrapped the bus, started walking there, walking back, nae matter what the weather – I was determined to dae it, and aye, it’s helped us. |
| Natasha | |
| Neil | |
| Sarah | I’ve joined Weight Watchers a couple of months ago … |
| Anne | As I got older I kinda like joined slimming clubs and stuff like that. … , I got a lot bigger just when I was about 20, about 19/20, and I decided that’s it for my 21st I gonnae lose it all, and I joined a slimming club. |
| Elizabeth | |
| Kirsty | I’ve started Slimming World … just two weeks ago aye. |
| Lisa | I went to Weightwatchers classes and lost a good bit of weight. … the reason I left was a lot of it was getting me down because, em, there was too much emphasis on figures, like you’ve lost or you’ve gained or you’re this or you’re that. And I got so bogged down in it that, I thought, “God sake you know it’s gonna be the end of the world if I put on like, a pound, or half a pound”, when it really wasn’t because I was, leading a healthy lifestyle and, and eating well and all the rest of it. And so I had to separate myself from it. An’, Uni and dancing and drink made me happy for a few years. |
| Richard | My cousin dragged me tae Weight Watchers. …It’s actually alright. I liked it. I went for aboot four months …<br><br>I’ve got a family doctor … She’s always geeing me an earful to get oan at me, and every time I go up that’s the first thing she does. If I go up for a sore throat she weighs me, so she’s always on my back to get me to lose weight. …P: So I’ve no been up for aboot eight month noo, coz I’m terrified of going up again in case she shoots at me again. |

**Supplementary Table 5: Illustrative quotes according to participant 'slimmer', 'relapser', 'stable' and 'gainer' categorisation – young adult transitions**

| **SLIMMERS** | |
|---|---|
| Catherine | EDUCATION: I done my beauty therapy first, and then I done my hairdressing, so I knew, like, both ends aw it – and even in that, they gie you a, it's like a, a health routine. You know things that are healthy, things that are fattening, what this'll do to your body, what that'll do to your body ... And it kinda opens your eyes to things that you're eating and what it is doing to you …<br>WORK: I changed my jobs in August last year, and since then, the amount of weight I have lost is unbelievable. I think I've lost about a stone and a half since August …it's just through daen more, being more active, than compared to what I was doing.<br>PARENTHOD: I was like that, and "I want to be around to play with my wean and be able to do things like that as well". I was like that, "I can't do that if I'm damaged, through what I've been doing". So I just decided to get, cut it all down, and just stop half the things completely. So I'm a lot healthier, today, than what I was back then … |
| Nina | LEAVING HOME: I love my independenc3 and getting on with it myself. I exercise more, I eat better, I'm just a more confident person when I'm away from my, my family and away from [named town]. |
| Noel * | WORK: I climb every day. That's what I dae at work I'm a climber know, I climb trees. And maist guys that climb trees are dead skinny and they eat like horses you know so I think it is just the way I'm gonna be noo know. |
| Alan | WORK: [Notes only – Needed to improve fitness for admission to RAF so began running and cycling – didn't lose weight but increased muscle mass and lost fat.] |
| Charlie | EDUCATION: And then I dunno if it was coming o' age or, it was just that I started going to University, was it four years ago now and eh, started going to the gym. Then that's the reason- well that's the only reason why I've lost- lost- I lost like four stone. Just going to the gym. |
| Clare | EDUCATION: But then start of Uni and things like that, cos it had a gym in it, I used to use that all the time. … Probably just got me into a habit … |
| Eilidh | EDUCATION: … realised that I was going to Uni, I didn't want to be big, it was like a new kinda fresh start. And so when I was probably when I was about 17½ I decided just to start healthy eating, cos I, as I said I'd try to go onto like step classes and stuff and it never really worked for me so I just started really healthy eating and really watching what I was doing. … since I started Uni I've always been into swimming, I love going swimming. So that's that. |
| Emma | WORK [POLICE TRAINING/WORK]: Well, em, I go the gym, obviously, because, I'm not saying because of my job, that sounds awful, but it is. … when I joined, I was nineteen when I joined, and I'd gone, I'd gone down to ten and a half stone, and they told me I still had to lose more weight, …, it is the most active I think I have ever been in my, in my life. Em, you're just constantly getting, and that's the reason why I had obviously kind of came down to like a size ten. |
| Janine | WORK: most of the, the jobs that I was doing was dancing and singing so I was in the gym all the time, and I was dancing in shows every night, but then there's the social side of it as well, where, coz you're only working for an hour a night, you used to go out drinking on the night time and you'd have all day to recover, so it was a bit of both – bad and good with that. |
| Mark | EDUCATION: just coming to uni was the sort of the biggest change ever … the start of my second year, that was really when things changed, like a… just everything changed, I guess. … I joined the sports union, so I joined some sports clubs, so I got my gym memberships so I started going there. I started working for [named local council] and started coaching, which kept me really active and was really good, and one of the guys that I was working with was a strength conditioning instructor, and he would sort of, he invited me into the gym a few times. … it became part of my life and it has remained so to this day. … I feel in myself that I really flourished in that environment from being a kind of uncomfortable little chubby kid, to being somebody who actually feels alright and, and is quite enjoying things. And yeah, uni was the real kind of… yeah, real turning point, definitely.<br>LEAVING HOME: luckily, probably it all coincided at just the right time because I had to start buying my own food …I was able to take control a bit more … |
| Pete | LEAVING HOME: Well I just left home about four or five months ago aye so … the first, first month or so I was in here, in the flat my diet was terrible. I think I probably put on about half a stone in a month. Which is terrible and I've, I've you know actually followed a Weight Watcher's diet for a few weeks aye, and I lost, I think I lost about ten pounds in three weeks |
| Rachel | |

| | |
|---|---|
| Scott | EDUCATION: The lifestyle wasn't so much a big thing about until I turned maybe eighteen, nineteen and started doing my degree then I started learning how to use a gym properly and what sort of exercise that I can do and just I'm now very aware of cos I'm working in nutrition what it is I actually take in and what it is I actually expend. |
| **RELAPSERS** | |
| Colin | WORK: And now that I'm putting on the weight that I, especially now, coz I'm working aw the time, and I'm eating in my work and then I'm coming home and having my dinner, em … I need to figure out a way where I can be more, like, active, rather than coming home and just sitting watching TV there, for the rest of the night., |
| Laura | |
| Malcolm | LEAVING HOME: then moved in here. It was like, we're not eating, Neil was like that "we're not eating, processed food outa box we're gonna make our dinners" and that's when I was like, "oh fair enough". |
| Patricia | EDUCATION: when I was seventeen and I went to uni, I was getting up at half six in the morning and I was having to skip breakfast, going to uni, being in lectures all day, having to go to the library where you're not allowed to eat or drink, and type up essays and things, and I did lose like I say, I drastically halved my body weight … |
| Patrick | WORK: I worked up at the sport centre as a lifeguard so I was really into swimming at that time and a bit of running as well. … and obviously the attraction o' it being free once you worked there you get the, the facilities free so that was always a bonus and watching, you could see, when you're on duty you could see like a swimming club and that and that gave me a bit of a drive to take it on, take it a bit further and take it a bit more seriously and then I done a few sponsored swims and that for charity. So that's, it's probably the fact that it was free, that's the main thing that it got me right into it. |
| Philip | WORK: I think I then lost weight, … not deliberate changes but I suppose em… for example the- the summer in between em… in between sixth year and going to University I was working full-time. And, so I didn't have- I wasn't able to go to like Gregg's twice a day and stuff like that.<br>LEAVING HOME: Eh more recently em, I suppose I've, I've occasionally, not always but occasionally made a concerted effort to choose healthier foods and things like that. Which hasn't- which was never really a concern until I moved away from home and that- then aye, it's since I started like buying my own shopping and doing ma own cooking. |
| Donna | EDUCATION/LEAVING HOME: That wasn't actually so much of a help because I was living on my own. At student houses and everything else and takeaways was a much more tempting option than cooking for yourself more often than not. Again throughout my Uni career, first to fourth year, I gradually, I definitely improved. I got a grip of that and decided that eating healthy was, was the best option so I started cooking for myself. |
| Geoff | WORK: So sometimes you canny be bothered daeing anything when you come in fae work. Well I canny anyway, I don't know aboot everybody else but I would sit and watch the telly or play the computer. |
| STABLE | |
| Chris | EDUCATION/LEAVING HOME: When I was at uni and I joined the gym and pretty much spent all the money I had on cigarettes and alcohol and didn't eat as much as probably I should have, but not in a you, know, not in a deliberate way, just like I used to never have any money for food and so I lost quite a lot of weight then. … so it was always kind of like, well I've got some rice and some beans in the cupboard so you know I can eat, so I may as well go and buy cigarettes instead because they make me happy but yeah, so… but that, I mean, that's obviously that was… I think, in a funny way, kind of good for me because I had quite a big sort of drop in my weight from what I'd been like between eighteen and nineteen. |
| Christina | LEAVING HOME: Think it was movin' in here, movin' intae the house that kinda done it for me, do you know what I mean? I kinda grew up, do you know what I mean? Realised that I had tae dae somethin'. I don't know I think it was by accident, it was. … I honestly dae I don't think I went oot my way tae lose any weight, do you know what I mean? It just kinda happened for me. Em. So I'd cut doon on the junk food and stuff like that and I was doin' more, do you know what I mean? …Like when I first moved in here, I used tae go walkaboot. I didny like stayin' myself, that's wan aw the reasons that we had people in livin' with me. But I got tae the stage where see when they wereny here, I wasny sittin' in the hoose myself, so I'd go out, do you know what I mean? |
| Jenny | WORK: The job I'm doing now … it's like eight hours of power walking, pushing this massive trolley. And I've been doing that for like five months now and I've noticed a difference, you feel a lot better doing that. …just for getting a lot more exercise than that, rather than sitting at a desk all day or something. |
| GAINERS | |

| | |
|---|---|
| Jamie | EDUCATION: There was first, first and second year at Uni when I just, you know I discovered you know booze. And then that really was us off to the races in terms of overweight. At one point, I, I drank way too much. My diet got even worse. … Getting no exercise and I ballooned. And I really, I went from being an overweight teenager. Well it wasn't like you could just say I was a little, I was fat at school and I really, really got huge I mean I probably, I think I was at 17½, I was about 17½ stone at my heaviest. I, I was that, I was that big. |
| Matthew | WORK: These days I work, in a much more stressful and- and- and long working environment. That em, after a day's work I'm absolutely knackered. And I don't wanna go out for a run. |
| Michael | WORK: … so we bought the treadmill and the, the multigym and the stepper and aw that, and stuff, so I wasnae working when I was at college, so I had plenty time to be daen it – but noo I'm in full time work, and I like my weekends – so I'm no' gonna spend them in the gym. |
| Natasha | |
| Neil | WORK: I was labouring for a wee while. I must have laboured for about six months … But the thing wae work was, because I seen the difference in my health, straight away – I didn't try to lose weight, when I started the job, I didn't try to lose weight, initially, at all – it didn't enter my mind. I didn't think I'd have lost it, 'cause I think I just thought 'I've gotta build up', em, but because I was labouring, very, eh, intense workout, basically every day, but you don't realise because it's just your work. … then it became, for me, at my work, at my workplace, where I could be getting paid for losing weight, basically. |
| Sarah | WORK: I've got a job, I'm a nurse so I can't really sort of preach healthy living to people if I'm not actually doing it myself I suppose. |
| Anne | EDUCATION: … just as I turned eighteen, I'd just thought I wanted to be like every other student and not have to what everyone says. Probably my diet wasn't the healthiest then but, probably six months later I thought right, that's when I joined like my first slimming club. |
| Elizabeth | |
| Kirsty | WORK: [shift-work] it was when I started working in there that I really put weight on, it was just, you know, getting up in the morning, and going to work and it would be, you know, no eating breakfast again, and grabbing a bar of chocolate, or during the day if we were sitting, somebody would maybe nip oot tae the shops and come back wi' sweeties an' you know, and being in the office your no getting that much exercise, and, so it's no a very healthy environment … I was coming in and getting a lot of takeaways and things, or microwave food you know that I could just, throw in when I got in from work or, or if I was at work, maybe if I was there late a microwave meal there, so, it wisnae very healthy food I was eating, no a lot of home cooked stuff, it was aw convenience food. |
| Lisa | EDUCATION: between sort of fifteen and, nineteen I would say, I was on quite a- not a strict diet but a very healthy diet. And I had- was losing weight, and was quite hap-happy, healthy and active. And then I went to uni and it all went downhill. As it does, too much drink and too much people saying, c'mon we'll just go to MacDonald's cos we're hungover and- not so good.<br>WORK: I think I'm getting that fitness back because there was a period I was out of work an'- and just before I was out of work, I was in a job that I sat down. So I lost a lot of my fitness there … |
| Richard | EDUCATION: … during college, I lost a lot of weight, probably, I lost aboot four stone, coz all of a sudden, after high school, I ballooned up while I was working, and then I went to college again, I lost it aw again – and then after college, it aw went back on again (laughs). But so I mean, during college I was fit and healthy and I was playing football every day and going to the gym. … My tutor told me to gie, gie healthy eating a go and all that. When I went back, it was a six month course at first, and he says, when I came back, he said I should try and lose a wee bit of weight, coz obviously you shouldnae be going into high schools and teaching kids aboot healthy eating and everything if you're the size of a, well he never quite put it that way, but that's what he was basically saying. |
